# Oxidative Stress Induced by Pt(IV) Pro-drugs Based on the Cisplatin Scaffold and Indole Carboxylic Acids in Axial Position

**DOI:** 10.1038/srep29367

**Published:** 2016-07-11

**Authors:** Dina Tolan, Valentina Gandin, Liam Morrison, Ahmed El-Nahas, Cristina Marzano, Diego Montagner, Andrea Erxleben

**Affiliations:** 1 School of Chemistry, National University of Ireland, Galway, Ireland; 2 Department of Chemistry, Faculty of Science, El-Menoufia University, Shebin El-Kom, Egypt; 3 Department of Pharmaceutical and Pharmacological Sciences, University of Padua, Italy; 4 Earth and Ocean Sciences, School of Natural Sciences and Ryan Institute, National University of Ireland, Galway, Ireland; 5 Department of Chemistry, National University of Ireland, Maynooth, Ireland

## Abstract

The use of Pt(IV) complexes as pro-drugs that are activated by intracellular reduction is a widely investigated approach to overcome the limitations of Pt(II) anticancer agents. A series of ten mono- and bis-carboxylated Pt(IV) complexes with axial indole-3-acetic acid (IAA) and indole-3-propionic acid (IPA) ligands were synthesized and characterized by elemental analysis, ESI-MS, FT-IR, ^1^H and ^195^Pt NMR spectroscopy. Cellular uptake, DNA platination and cytotoxicity against a panel of human tumor cell lines were evaluated. All the complexes are able to overcome cisplatin-resistance and the most potent complex, cis,cis,trans-[Pt(NH_3_)_2_Cl_2_(IPA)(OH)] was on average three times more active than cisplatin. Mechanistic studies revealed that the trend in cytotoxicity of the Pt(IV) complexes is primarily consistent with their ability to accumulate into cancer cells and to increase intracellular basal reactive oxygen species levels, which in turn results in the loss of mitochondrial membrane potential and apoptosis induction. The role of the indole acid ligand as a redox modulator is discussed.

The discovery of cisplatin and its approval by the FDA in 1978 has led to the development of the second and third generation platinum drugs carboplatin and oxaliplatin. Today, cisplatin, carboplatin and oxaliplatin are the most important anticancer drugs used in chemotherapy (Fig. 1)^1^,^2^,^3^. These three platinum(II) complexes are believed to operate by the same mechanism of action by undergoing hydrolysis to form the reactive aqua species which are responsible for the binding to purine bases in DNA to form DNA adducts and to trigger apoptosis^4^,^5^. Despite the clinical success of Pt(II) drugs, their usage is limited due to their drawbacks, mainly their high toxicity and severe side effects which arise from a lack of selectivity and their undesirable reactions with biological nucleophiles and proteins^6^,^7^.

One of the approaches to overcome the drawbacks of Pt(II) drugs is to use Pt(IV) complexes^8^,^9^,^10^. Octahedral Pt(IV) complexes can serve as pro-drugs that are activated by intracellular reduction to the corresponding Pt(II) agents^11^. Pt(IV) compounds are used to overcome the limitations of Pt(II) drugs by resisting premature aquation and unwanted binding to essential plasma proteins^12^. They have shown considerable promise for both oral administration and for reducing side effects. The most likely reason why Pt(IV) complexes such as satraplatin can be administrated orally–in contrast to Pt(II) drugs which are delivered intravenously–is the kinetic inertness of Pt(IV) complexes due to their low spin d^6^ electronic configuration^5^. Pt(IV) complexes usually undergo reduction inside the cell by biological reducing agents (e.g. ascorbate, glutathione) to the active Pt(II) species before binding to their ultimate target DNA (Fig. 2)^13^. The reduction that is accompanied by the loss of the axial ligands has been extensively studied^14^. Mechanisms proposed for the reductive elimination include outer-sphere mechanism, inner-sphere mechanism and Pt(II)-catalyzed reaction pathways. The latter involving bridged Pt(II)-Pt(IV) dimers seems unlikely under cellular Pt complex concentrations. The reduction potential of cis,cis,trans-[Pt(amine)_2_X_2_Y_2_] complexes is primarily determined by the axial ligands and typically follows the order Y = Cl > COOH > OH. Contrary to what would be expected, the reduction rate of Pt(IV) complexes does not always correlate with the reduction potential. Chlorido and hydroxido ligands can serve as electron bridges facilitating the faster inner-sphere electron transfer between ascorbic acid or thiol and the Pt center. By contrast, carboxylate ligands are poor electron transfer bridges and their complexes are generally reduced more slowly^15^,^16^,^17^,^18^. However, there is little clarity on the reduction rates, products and reducing agent *in vivo*. Recent work by Gibson and coworkers has suggested that the *in vivo* reduction of Pt(IV) complexes is chiefly mediated by biomacromolecules rather than by small-molecule reducing agents such as ascorbate^19^. Furthermore, there is growing evidence that the equatorial ligands also play a role and in some cases they can be released complicating the intracellular fate of Pt(IV) complexes^20^,^21^.

Besides for tuning the reduction potential and thus the stability, the axial ligands of Pt(IV) pro-drugs can be used to modify other important pharmacological properties like lipophilicity. Furthermore, the axial coordination sites can serve as binding sites for other biologically active ligands^22^,^23^,^24^,^25^,^26^. This class of platinum complexes is of particular interest for the design of dual-threat pharmaceutical agents, which combine two biologically active components into a single molecule^27^,^28^,^29^.

Pt(IV) compounds that have recently been investigated in this context include complexes with the histone deacetylase inhibitors valproate and phenylbutyrate as the axial ligands as well as with dichloroacetate which is a known apoptosis sensitizer and anticancer agent reverting the Warburg effect^30^,^31^,^32^. Recently, Pt(IV) derivatives of cisplatin with the vitamin E analog α-tocopheryl succinate (α-TOS) as axial ligands were shown to exhibit a cytotoxic activity up to 25 times greater than that of cisplatin across several tumor cell lines, being able to induce nuclear DNA damage and simultaneous mitochondrial membrane depolarization^33^.

Here we describe the synthesis, characterization and biological properties of novel Pt(IV) complexes as cisplatin pro-drugs with indole-3-acetic acid (IAA) and indole-3-propionic acid (IPA) as biologically active axial ligands. Indole compounds are receiving considerable attention as redox modulators^34^,^35^,^36^,^37^,^38^. They have antioxidant and radical scavenging properties. IPA is known to protect against the oxidation of lipids^39^,^40^ and has been shown to reduce the autoxidation and iron-induced lipid peroxidation in hamster testes^48^. On the other hand, indole carboxylic acids can be oxidized to cytotoxic species. IAA, for example, can act as a non-toxic pro-drug that forms radicals when activated by redox catalysts such as horseradish peroxidase (HRP)^40^. This in turn can lead to enhanced cellular oxidative stress. Indeed, IAA is used in the oxidation therapy of cancer and for the treatment of Acne Vulgaris^33^,^34^.

The possibility of Pt(IV) complexes inducing the formation of reactive oxygen species (ROS) has been considered only recently^41^,^42^. Because of metabolic and signaling aberrations, cancer cells already have elevated ROS levels and the induction of further oxidative stress has been recognized as an attractive strategy to selectively kill cancer cells^43^. On these bases, herein we investigated the potential antitumor activity of new Pt(IV) complexes bearing IAA and IPA as axial ligands, focusing our attention on ROS production and mitochondrial membrane potential perturbation. We hypothesized that the interplay between the redox active Pt(IV) metal center and the indole carboxylic acid ligands may severely unbalance cellular redox homeostasis, thus potentiating the cytotoxic effect of the Pt complexes.

## Results

### Synthesis and Characterization

**Figure 3:**
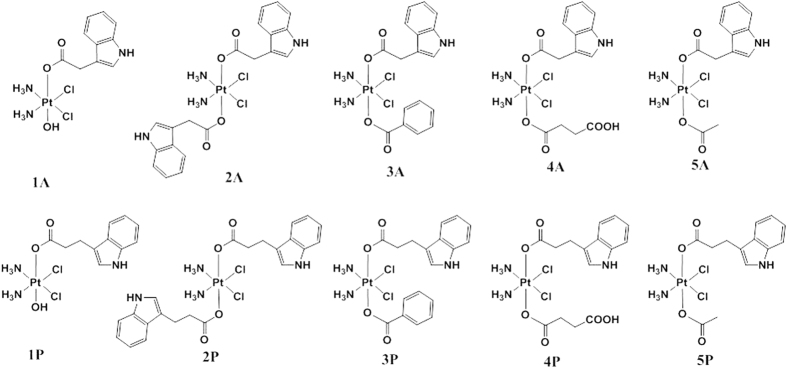
Structures of the ten Pt(IV) complexes described in this paper.

Ten indole carboxylic acid Pt(IV) complexes with IAA (indole-3-acetic acid, **1A**–**5A**) and IPA (indole-3-propionic acid, **1P**–**5P**) were synthesized and the structures are depicted in Fig. 3. The monocarboxylato complexes **1A** and **1P** were prepared by reacting oxoplatin, c,c,t-[Pt(NH_3_)_2_Cl_2_(OH)_2_], with 0.8 eq. of the activated NHS-ester of the corresponding indole carboxylic acid. The dicarboxylato complexes with two indole acids in axial position (**2A** and **2P**) were obtained using an excess of the NHS-ester activated acid (20 eq.), since the second hydroxo group of oxoplatin is much less reactive than the first one. The unsymmetrically substituted complexes (**3A**–**5A** and **3P**–**5P**) were prepared by reaction of the monocarboxylate complexes **1A** and **1P** with the corresponding anhydrides. We chose three different anhydrides (benzoic, acetic and succinic anhydride) to vary the lipophilicity of the resulting Pt(IV) complexes. All the synthesized Pt(IV) complexes were characterized using elemental analysis, ESI-MS, FT-IR, ^1^H and ^195^Pt NMR spectroscopy. The purity of the complexes has been assessed through elemental analysis and–where solubility was not an issue–by high performance liquid chromatography (HPLC). The dicarboxylato complexes are poorly soluble in organic solvents. The proton NMR spectra of the complexes in DMSO-*d*_*6*_ show the typical signal of the NH_3_ protons which is a multiplet at around 6 ppm for the monocarboxylato complexes **1P** and **1A** (typical ^1^*J*_NH_ and ^2^*J*_PtH_ values around 50 Hz) while it is a broad signal around 6.5 ppm for the bis-substituted complexes (**2**–**5A** and **2**–**5P**, Supplementary Information)^44^. The mass spectra of the complexes show a peak in the negative mode with the typical platinum isotope pattern corresponding to M − H^−^ or M + Cl^−^. Stability studies on representative examples of the complexes (**1A**, **1P**, **3A** and **3P**) *via* mass spectrometry confirm that the complexes are stable in physiological saline (0.9% NaCl) for at least 48 h (Supplementary Information).

**Figure 4:**
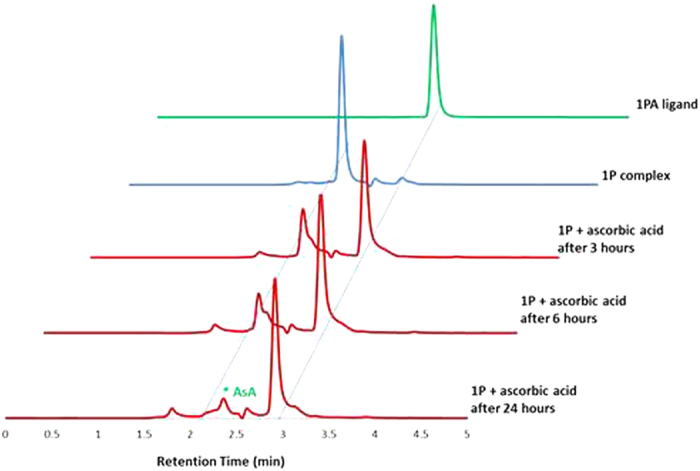
HPLC chromatogram of the reaction of 1P with 10 eq. ascorbic acid (AsA) at 37 °C and pH 7. The chromatogram of the free ligand (IPA) is shown for comparison purposes.

**Figure 5:**
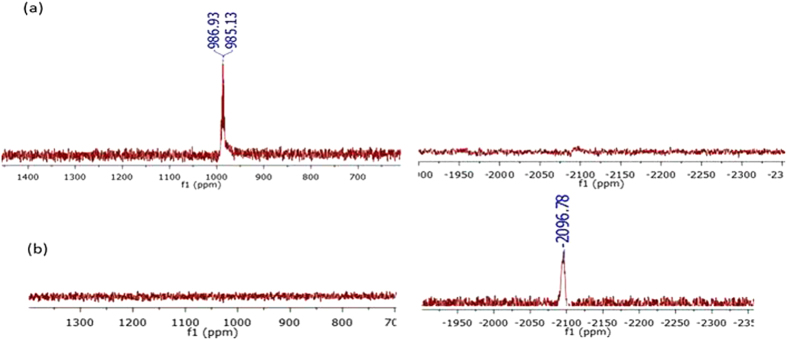
^195^Pt NMR spectra of (**a**) complex **1P** and (**b**) after reacting **1P** with ascorbic acid for 24 h at 37 °C. Zooms of the 600 to 1400 and −2300 to −2000 ppm regions are shown.

The reduction reaction of two representative complexes (**1A** and **1P**) and the release of the indole carboxylic acid ligands was analyzed by HPLC using ascorbic acid as the reductant and 37 °C, pH 7 as physiological conditions (Fig. 4 and S1). The HPLC chromatograms of the reduction reaction of the complexes were compared with the chromatograms of pure samples of the carboxylic acid ligands. The results indicated that the complexes release the indole carboxylic acid ligands upon reduction from Pt(IV) to Pt(II). After 3 h incubation with 10 eq. of ascorbic acid 75% of **1P** and 50% of **1A** was reduced. Complex **1P** was completely reduced after 24 h, while complex **1A** was completely reduced after 30 h. We further studied the nature of the Pt(II) species produced upon the reduction of **1P** and **1A** by ^195^Pt NMR. Figure 5 and S2 show that the reaction of **1P** and **1A** with 1 eq. of ascorbic acid resulted in a strong signal for cisplatin at −2096 ppm^45^,^46^. The complexes were completely reduced after 24 h (**1P**) and 30 h (**1A**) of incubation with ascorbic acid at 37 °C, in line with the HPLC analyses. Unfortunately, monitoring the reduction reaction of the symetrically (**2A** and **2P**) and unsymmetrically (**3A**–**5A** and **3P**–**5P**) substituted dicarboxylato complexes was hampered by the poor solubility of the complexes in water and most organic solvents except DMF and DMSO. For the same reason the reduction potentials could not be determined by cyclic voltammetry. However it has been reported that the reduction rate of cis,cis,trans-[Pt(NH_3_)_2_Cl_2_(RCOO)(OH)] monocarboxylato complexes is faster than that of dicarboxylato complexes and this has been attributed to the fact that hydroxido ligands are much better bridging ligands for electron transfer than carboxylate ligands^45^.

It is known that the activity of cisplatin is mainly due to DNA damage caused by its ability to bind to the N7 position of guanine bases. To mimic the intracellular reduction, complex **1P** was reduced with sodium ascorbate in the presence of guanosine to simulate the interaction of the platinum drug with DNA *in vitro*. The reduction of **1P** followed by the reaction with guanosine (G) led to the formation of the Pt^II^/GG bisadduct [Pt(NH_3_)_2_(G-*N7*)_2_] (m/z 795 in the mass spectrum, Figure S3, Supplementary Information). This suggests that cisplatin formed upon reduction of **1P** will interact efficiently with nuclear DNA.

### Cytotoxicity

The cytotoxic activity of the complexes and of the uncoordinated ligands was evaluated by the MTT test on a panel of human tumor cell lines containing examples of pancreatic (BxPC3), colorectal (HCT-15), breast (MCF-7), cervical (A431), and lung (A549) cancers as well as of melanoma (A375). For comparison purposes, the cytotoxicity of cisplatin was evaluated under the same experimental conditions. IC_50_ values, calculated from the dose-survival curves obtained after 72 h of drug treatment, are shown in Table 1. The uncoordinated ligands proved to be hardly effective against all tested cancer cell lines, whereas the newly synthesized Pt(IV) complexes were able to promote a significant cancer cell killing effect. Noteworthy, they were extremely effective against human lung A549 cancer cells, notoriously poor sensitive to platinum drugs, being on average 2.4-fold more cytotoxic than cisplatin. Among all complexes, the monohydroxido complex with indole propionic acid, **1P**, showed the highest cytotoxic activity, with a mean IC_50_ value of about 2.5 μM, roughly three times lower than that calculated for cisplatin (mean IC_50_ = 8.33 μM). Similarly, the unsymmetrically dicarboxylated complex **3P** with benzoic acid exhibited a cytotoxicity profile comparable to that of the derivative **1P**, with an average IC_50_ value of 2.6 μM. By contrast, compounds **4A**, **4P** and **5A** appear to be the least cytotoxic in all the considered cell lines, with mean IC_50_ values up to one order of magnitude higher than those calculated for **1P** and up to nine times higher than those calculated for cisplatin. In general, regarding the indole propionic acid series, the monocarboxylated Pt(IV) complex **1P** was more effective than the dicarboxylato Pt(IV) derivative. Indeed, the introduction of a second carboxyl ligand to the other axial position, forming the symmetrically disubstituted derivative **2P**, led to a 50% decrease of cytotoxic activity (mean IC_50_ equal to 2.5 and 4.9 μM for **1P** and **2P**, respectively). The introduction of a second and different carboxyl ligand (benzoate, acetate or succinate) in axial position led to Pt(IV) derivatives for which the activity profile depends on the nature of the substituent. More precisely, the complex bearing the most lipophilic benzoate ligand, **3P**, showed the highest *in vitro* antiproliferative activity (mean IC_50_ equal to 2.6 μM), whereas the complexes with a more hydrophilic carboxylate, such as succinate and acetate, **4P** and **5P**, were endowed with a lower cytotoxic activity (mean IC_50_ of 30.4 and 11.1 μM, respectively). A rather similar behavior occurred with the Pt(IV) complexes with indole acetic acid in axial positions. Again **1A**, which presents a single axial carboxylate ligand, appeared to be the most active compound, with mean IC_50_ values about 2.5 times lower than those obtained with the reference chemotherapeutic metallo-drug. Coherently, when a second and different axial carboxylate ligand was introduced, the activity correlated with the lipophilicity of this ligand, i.e. **3A** > **5A** > **4A**.

It is important to notice that the most cytotoxic derivatives **1A** and **1P** were about two times (in HCT-15 cells) and four times (in BxPC3 and A549 cells) more effective than cisplatin in inhibiting cancer cell proliferation.

### Cross-resistance studies

The cytotoxic properties of the complexes were also evaluated in a pair of tumor cell lines suitably selected for their sensitivity and resistance to cisplatin (adenocarcinoma cells 2008/C13^*^). Cross-resistance profiles were evaluated by means of the resistance factor (RF), defined as the ratio between IC_50_ values calculated for the resistant cells and those obtained with the sensitive ones (Table 2). The phenomenon of cisplatin resistance is multifactorial in nature. Nevertheless, the main molecular mechanisms involved in drug resistance in C13* cancer cells have largely been identified. Indeed, in this cell line resistance is correlated to high cellular glutathione and thioredoxin reductase levels^47^, reduced cellular drug uptake and enhanced repair of DNA damage^48^. All derivatives demonstrated a similar cytotoxic profile, both in cisplatin-sensitive and -resistant cell lines, with a cytotoxicity pattern highly similar to that described above, with complexes **1A** and **1P** again being the most active complexes in the two series. The RF values calculated for all derivatives were six- to nine-fold lower than that of cisplatin, clearly indicating the absence of cross-resistance.

### Cellular uptake and DNA platination

**Figure 6:**
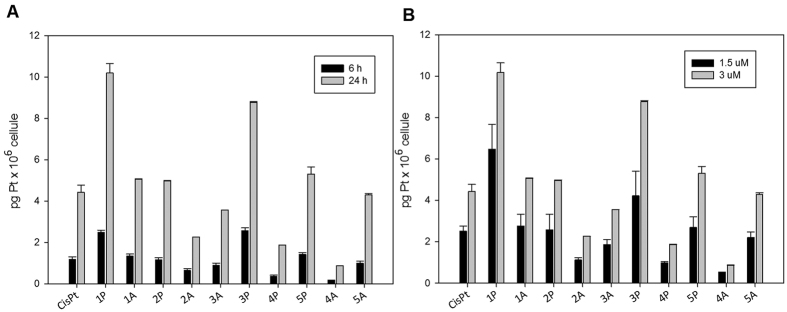
Cellular uptake of Pt(IV) complexes in A375 melanoma cells. (**A**) A375 cells incubated for 6 and 24 h with the complexes at 3 μM concentration. (**B**) A375 cells incubated for 24 h with the complexes at 1.5 and 3 μM concentration. The intracellular platinum content was determined by GF-AAS.

One of the key factors determining the biological activity of a metal-based compound is its ability to cross cell membranes and to accumulate into cancer cells. Therefore, in order to correlate the antiproliferative effect shown by the complexes and their cellular accumulation, the platinum content was evaluated in A375 cells following treatment with equal doses (1.5 and 3 μM) of the tested compounds for 6 and 24 hours. The intracellular metal amount was quantified by means of graphite furnace atomic absorption spectroscopy (GF-AAS), and the results, expressed as pg of metal per 10^6^ cells, are shown in Fig. 6, panel A and B. Although to a different extent, all complexes accumulated proportionally to their concentration and in a time-dependent manner. Among all derivatives, **1P** and **3P** were internalized most efficiently. In particular, following 24 h exposure, the amount of platinum detected in cells treated with **1P** at 3 μM concentration was about 2.3 times greater than that observed in cells treated with the same dose of cisplatin. By contrast, **4A** and **4P** gave the lowest platinum accumulation in treated cells, with cellular uptake levels about 7 times lower compared to cisplatin.

**Figure 7:**
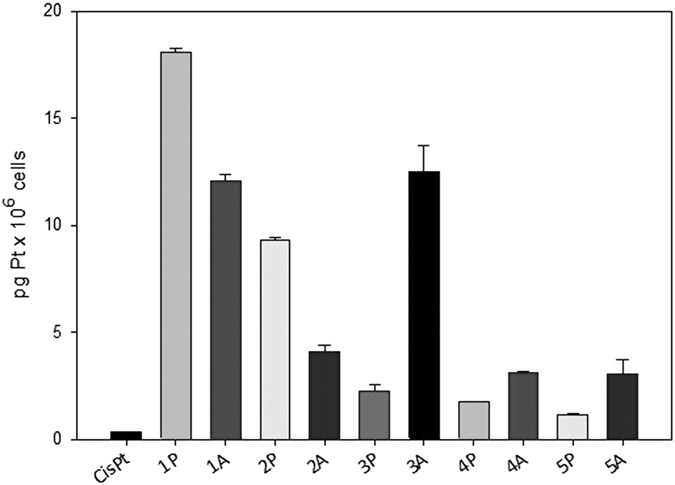
Nuclear DNA platination levels in A375 cells treated for 24 h with 5 μM of the tested complexes. The amount of Pt in each sample was determined by means of GF-AAS analysis.

It is well known that the main cellular target of platinum complexes is the DNA, and platination of nuclear DNA is a critical step in triggering the death of cancer cells by platinum-based drugs. On this basis, the amount of platinum bound to nuclear DNA was also assessed in A375 cells treated with the tested Pt(IV) complexes. Cells were treated for 24 h with 5 μM of the tested complexes, the nuclear DNA was isolated and the amount of Pt in each sample was determined by means of GF-AAS analysis. The results expressed as pg Pt/ng DNA are depicted in Fig. 7. Notably, all Pt(IV) complexes elicited a degree of DNA platination significantly higher than that of cisplatin. Among all tested compounds, the least effective ones in interacting with cellular DNA were the IPA derivatives **4P** and **5P**, whereas compound **1P** led to the highest degree of DNA platination. In particular, **1P** led to a roughly 60-fold higher DNA platination level with respect to cisplatin. Considering the IAA series, the derivatives **1A** and **3A** led to the most effective platination of cellular DNA, whereas **4A** and **5A** were endowed with the lowest DNA platination capability.

Partition coefficients (log*P* values) for octanol/water partition were determined in order to examine whether a correlation exists between lipophilicity and accumulation into cells. The log *P* values obtained (Table 3) give the trend in lipophilicity as mono indole acid complex < succinate complex < acetate complex < bis indole acid complex < benzoate complex for both the IAA and the IPA series.

### ROS production

**Figure 8:**
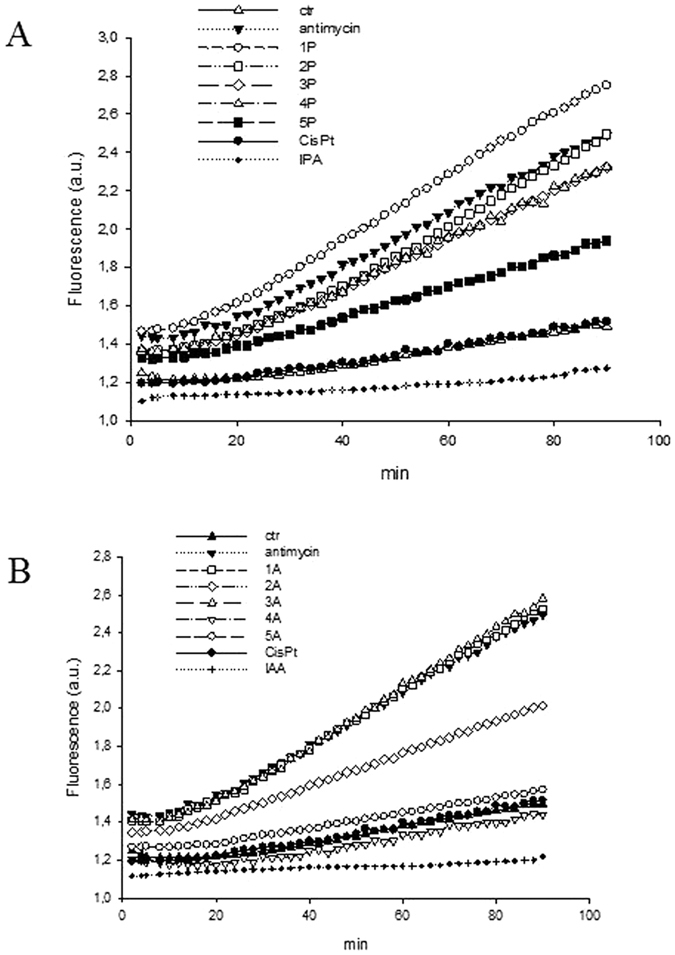
ROS production in A375 cells. Cells were preincubated in PBS/10 mM glucose medium for 20 min at 37 °C in the presence of 10 μM CM-DCFDA and then treated with equimolar doses (12.5 μM) of indole propionic acid Pt(IV) derivatives (panel A) or indole acetic acid Pt(IV) derivatives (panel B). The fluorescence of DCF was measured.

ROS are products of normal cell metabolism but their levels can be increased upon exposure to toxic agents or interference with the cellular redox network. Notably, as described in the introduction, indoles in the presence of HRP or other redox catalysts, can be transformed intracellularly into pro-oxidants, able to unbalance the cellular redox state and provoking an increase in oxidative stress^40^. Consequently, cellular ROS levels upon treatment with equal doses (12.5 μM) of the different platinum complexes were monitored in A375 cancer cells by using the peroxide-sensitive fluorescent probe CM-H_2_DCFDA (5-(and-6)-chloromethyl-2′,7′-dichlorodihydrofluorescein diacetate, acetyl ester). Antimycin, a classical inhibitor of the mitochondrial respiratory chain at the level of complex III, was used as a positive control. The results, expressed as arbitrary units of fluorescence as a function of time, are shown in Fig. 8.

Interestingly, all IPA complexes were able to substantially stimulate the production of hydrogen peroxide in a time-dependent manner (Fig. 8, panel A), while the free IPA and IAA ligands in the absence of Pt had no effect on cellular basal ROS levels. In particular, **1P** induced an increase in ROS production that was superior to that induced by antimycin, whereas **2P** enhanced ROS production to a similar extent as the respiratory chain complex III inhibitor. Also the IAA complexes **1A** and **3A** were able to increase the H_2_O_2_ production to a similar extent as antimycin, whereas **2A** was less effective compared to the positive control (Fig. 8, panel B). Conversely, treatment of A375 cells with **4A** and **5A** did not alter the cellular ROS concentration.

### Mitochondrial membrane potential

**Figure 9:**
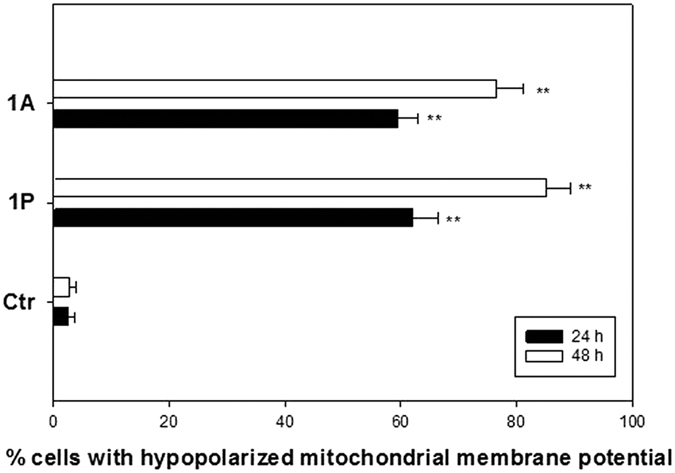
Effects of **1P** and **1A** on the cellular mitochondrial membrane potential. A375 cells were treated for 24 and 48 h with IC_50_ concentrations of **1P** or **1A** and stained with TMRM (10 nM). The percentage of cells with a hypopolarized mitochondrial membrane potential was determined fluorimetrically.

A persistent increase in the rate of ROS production is responsible for the accumulation of ROS. In turn, an increase in cellular ROS leads to the loss of the mitochondrial membrane potential (ΔΨm, MMP), thus possibly leading to the induction of apoptosis. The effect induced by the Pt(IV) complexes on the MMP was assessed by treating A375 cells with IC_50_ concentrations of **1P** and **1A** and evaluating the energization of treated melanoma cells in terms of the retention of the cationic fluorescent probe tetramethyl rhodamine methyl ester (TMRM). Compared to control cells, both derivatives elicited a significant and time-dependent increase in the percentage of cells with a decreased MMP (Fig. 9).

### Mechanism of cell death

**Figure 10:**
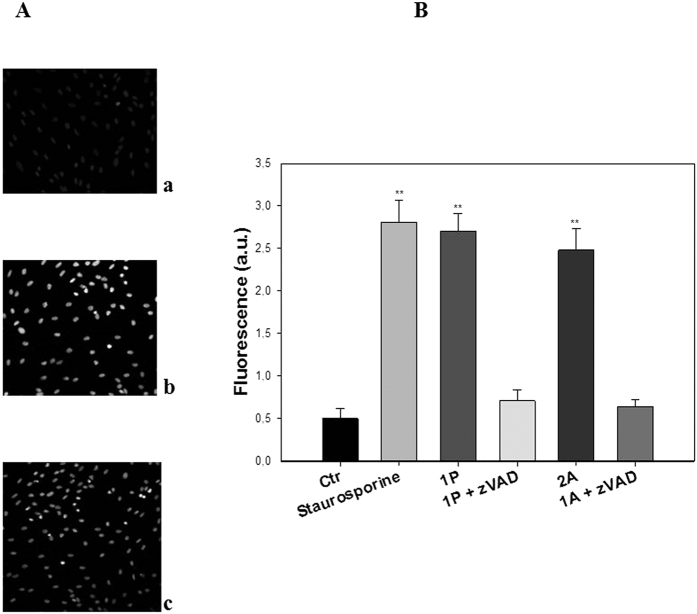
(**A**) Hoechst staining of A375 cells incubated for 48 h with IC_50_ doses of **1P** (**b**) and **1A** (**c**). Panel a represents untreated A375 cells as control. (**B**) Caspase-3 activity upon incubation of A375 cells for 48 h with IC_50_ doses of **1P** and **1A**.

In order to characterize cell death pathways activated in response to treatment with the most effective compounds **1P** and **1A**, we examined their effects in terms of apoptosis induction in A375 cells. Figure 10A shows the results obtained upon monitoring cellular morphological changes in A375 cells treated for 48 h with IC_50_ doses of **1P** and **1A** using Hoechst 33258 fluorescent staining analysis. Compared with control cells, treated cells presented brightly stained nuclei and morphological features typical of cells undergoing apoptosis, such as chromatin condensation and fragmentation.

Testing the ability of **1P** and **1A** to activate caspase-3, a well-known executor enzyme in the apoptotic pathway, it was found that both complexes markedly stimulated caspase-3 activity (Fig. 10B).

In particular, following a 48 h treatment with IC_50_ doses of **1P** and **1A**, the protease activity was enhanced roughly by a factor of 5 over untreated control cells. Caspase-3 cleavage reached values similar to that exerted by the well-known caspase-dependent apoptosis inducer staurosporine. Interestingly, pre-treatment of cancer cells with zVAD, a cell-permeable pan-caspase inhibitor, strongly decreased caspase activation induced by the Pt(IV) complexes, thus confirming the role played by these proteases in cell death caused by these derivatives.

## Discussion

Ten indole carboxylic acid Pt(IV) derivatives have been successfully synthesized and characterized. Among all the complexes, promising *in vitro* antitumor activity was found for Pt(IV) complexes based on the cisplatin scaffold, bearing one axial indole carboxylate and one axial hydroxido ligand (**1A** and **1P**). The cytotoxicity studies carried out on a large panel of human tumor cell lines have revealed that, generally, complexes with IPA (indole propionic acid) ligands were on average more cytotoxic than complexes with IAA (indole acetic acid) ligands. In particular, the monocarboxylated complex **1P** showed an antiproliferative activity approximately three times superior to that of cisplatin. In addition, all the complexes are able to overcome cisplatin-resistance. Platinum uptake experiments showed that all the Pt(IV) complexes are internalized inside cancer cells better than cisplatin, with **1P** being the ablest to accumulate into cells. Thus, noteworthy, the trend in cellular accumulation cannot be directly related to the lipophilicities of the complexes. Indeed, the most efficient internalization is observed for the least lipophilic complex **1P**. Although a higher lipophilicity should lead to an enhanced cellular uptake, as Pt(IV) complexes (unlike Pt(II) complexes) enter cells solely by passive diffusion, a lack of correlation between log*P* values and cellular Pt levels is not unprecedented^31^,^32^.

**Figure 11:**
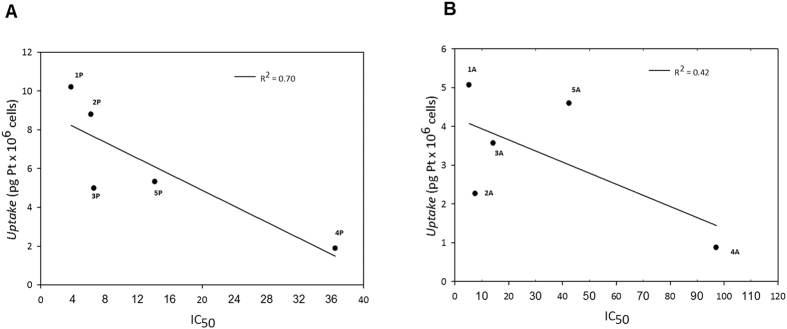
Correlation between cytotoxicity (IC_50_) and cellular platinum levels in drug-treated A375 cells. (**A**) propionic acid derivatives **1P**–**5P**, (**B**) acetic acid derivatives **1A**–**5A**.

By correlating the data of cellular uptake with those arising from the cytotoxicity studies following 24 h of treatment, a direct correlation between cytotoxic activity and cellular uptake is revealed, albeit to a different extent for the IPA (Fig. 11, panel A) and IAA series (Fig. 11, panel B). Actually, the inverse correlation between IC_50_ values and cellular platinum content was much more pronounced in A375 cells treated with indole propionic acid derivatives (R^2^ = 0.71) compared to results obtained with the indole acetic acid complexes (R^2^ = 0.42). However, by comparing the cytotoxicity results with DNA platination levels elicited by the Pt(IV) complexes, no linear correlation was found. Taken together, these data strongly support the hypothesis that different targets could account for the antiproliferative effects of these newly synthesized Pt(IV) compounds.

**Figure 12:**
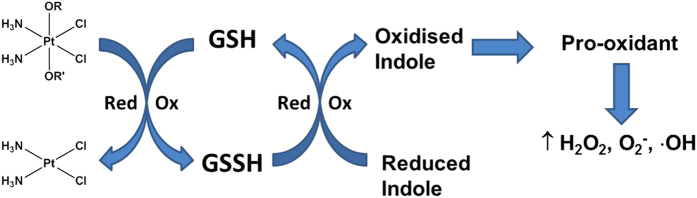
Proposed mechanism of ROS formation by Pt(IV) complexes with indole carboxylic acid ligands.

The higher potency of **1P** compared to the other derivatives thus appeared to be primarily due to its greater accumulation into cells and its ability to induce ROS formation. Actually, the most interesting result of the present study is the fact that some of the complexes were very efficient ROS producers. In particular, **1P** induced a dramatic increase in cellular ROS basal production, thus leading to a massive mitochondrial membrane potential loss. From a mechanistic point of view, we can speculate that, upon reduction of Pt(IV) to Pt(II), the released indole carboxylic acid undergoes oxidation and turns into a pro-oxidant species. In such a scenario, redox cycles could be triggered at the intracellular level that shift the redox state towards a more oxidized state (Fig. 12). In this context it is noteworthy that IAA and IPA alone did not elicit any significant cytotoxic effect.

Therefore, the introduction of redox modulators in the axial positions in a Pt(IV) complex can potentiate the antiproliferative effects through simultaneous DNA platination and ROS generation, both known triggers of apoptosis. The most potent complex, **1P**, is the most efficient ROS producer and gives the highest degree of DNA platination. Indeed, **P1** shows the highest level of loss of mitochondrial membrane potential and strongly enhances the caspase 3 activity (Fig. 9). Overall, the results of this study support the hypothesis that the Pt(IV) indole acid complexes have multifactorial targets.

## Conclusions

In summary, coordinating redox modulators to the axial positions of Pt(IV) complexes appears to be a profitable strategy for obtaining new promising anticancer agents. In particular, our work pointed out that the presence of indole acids in axial position of Pt(IV) prodrugs increases the production of ROS in the cellular environment, thus determining the loss of the mitochondrial membrane potential, ultimately leading to cancer cell death. This dual-threat mechanism combining the effects of DNA platination and ROS generation drives cancer cells to a caspase-dependent apoptotic cell death. In conclusion, the present study highlights the potential for developing Pt(IV) complexes with redox modulators as antitumor pro-drugs.

## Methods

### Materials and Syntheses

All reactions were carried out under normal atmospheric conditions. Solvents and chemicals were of analytical grade or HPLC grade and were obtained from commercial sources. The ultrapure water used was purified by a Milli-Q UV purification system. K_2_PtCl_4_ was obtained from Acros Organics. Indole-3-acetic acid, indole-3-propionic acid, succinic anhydride and acetic anhydride were obtained from Sigma-Aldrich. Benzoic anhydride, *N*-hydroxysuccinimide and dicyclohexylcarbodiimide were purchased from TCI. All chemicals were used as received. Cisplatin and oxoplatin were synthesized as previously reported^49^,^50^.

In general, the monocarboxylato complexes **1A** and **1P** were obtained by reaction of oxoplatin with 0.8 eq. of the corresponding activated NHS-ester of indole-3-acetic or indole-3-propionic acid in DMSO at 50 °C. The bis-indole acid complexes (**2A** and **2P**) were obtained in the same way using a ten-fold excess of the activated ligand in DMSO at 60 °C. The unsymmetrically biscarboxylated complexes were obtained by reaction of the corresponding monohydroxido species **1A** or **1P** with benzoic anhydride (**3A** and 3**P**), succinic anhydride (**4A** and **4P**) or acetic anhydride (**5A** and **5P**). Details on the syntheses and ^1^H, ^13^C, ^195^Pt NMR and mass spectra for all the complexes are reported in the Supplementary Information.

### Measurements

^1^H NMR and ^13^C NMR spectra were recorded on a Jeol ECX-400. Chemical shifts (δ) are reported in parts per million (ppm) and referenced internally using the residual solvent signals relative to tetramethylsilane (δ (^1^H NMR) = 0 ppm). Coupling constants *J* are reported in Hertz. One dimensional ^195^Pt NMR spectra were recorded on a Varian 500 AR spectrometer in DMF with insert D_2_O. K_2_PtCl_6_ in D_2_O was used as an external standard. Mass spectra were measured using a Waters LCT Premiere XE with electron spray ionisation and time of flight mass analyser. Elemental analysis (carbon, nitrogen and hydrogen) were performed by an Exeter Analytical CE-440. FT-IR spectra were recorded on a PerkinElmer FT-IR spectrometer fitted with an ATR accessory. A dynamic reaction cell ICP-MS (ELAN DRCe, Perkin Elmer, Waltham, USA), equipped with a flow injection autosampler (FIAS 93 plus) was employed for platinum determination^51^,^52^,^53^,^54^. Instrumental operating conditions were the following: ICP RF Power 1150 W: plasma gas flow 15 L min^−1^, auxiliary gas flow 1 L min^−1^, nebuliser gas flow 0.93 L min^−1^, observed isotopes, ^192^Pt, ^194^Pt, ^195^Pt, ^196^Pt and ^198^Pt). Calibration standard solutions were prepared from a single element standard (Inorganic Ventures, 1000 μg mL^−1^) prepared in Milli-Q™ water (18.3 mΩ) (Millipore, Bedford, USA) with 1% HNO_3_ (Trace Metal Grade, 67–69%, Fisher, UK). Iridium (^191^Ir) and indium (^115^In) were used as internal standards to account for instrumental drift and matrix effects. The HPLC studies were carried out using an Agilent 1200 series DAD analytical HPLC instrument.

### Reduction reaction studied by HPLC

The reduction of the complexes **1A** and **1P**) with ascorbic acid was followed by HPLC using a Phenomenex Luna C18 (5 μM, 100 Å, 250 mm × 4.60 mm i.d.) column at a flow rate of 1.0 mL/min with 280 nm UV detection at room temperature. The mobile phase was 80:20 acetonitrile (1% trifluoroacetic acid) : water (1% trifluoroacetic acid). The complexes were dissolved in DMF (0.5 mL) and added to a 5 mM solution of ascorbic acid in 2 mM 4-(2-hydroxyethyl)piperazine-1-ethanesulfonic acid (HEPES) buffer (pH 7) and diluted to a final concentration of 0.5 mM using acetonitrile. The process was followed at 37 °C until complete reduction.

### Reduction reaction studied by ^195^Pt NMR spectroscopy

Compounds **1A** and **1P** (10 mM) were incubated at 37 °C, respectively, in the presence of 10 mM ascorbic acid in 12 mM HEPES buffer at pH 7. The compounds were dissolved in DMF and then ascorbic acid and aqueous buffer solution was added (the concentrations given are the final concentrations). The reaction was followed until complete reduction of the complexes. The ^195^Pt NMR spectra were recorded by inserting a tube with D_2_O into the NMR tube.

### Reaction with guanosine studied by ESI-MS

Complex **1P** (1.5 mg, 3 μmol) and guanosine (4.24 mg, 15 μmol) was dissolved in 1 mL DMF and a 15 mM aqueous solution of sodium ascorbate (1 mL) was added. The mixture was kept at 37 °C for 24 h. Thereafter, the mixed solution was analyzed by ESI-MS.

### Determination of lipophilicity parameters

The log*P* values of the Pt(IV) compounds were determined using the shake flask method^55^. The respective Pt(IV) complex was dissolved in 0.9% NaCl (w/v) ultrapure water (presaturated with n-octanol). The solutions were sonicated and filtered to remove undissolved Pt(IV) compounds. The initial Pt concentrations were determined by ICP-MS. Subsequently, the Pt(IV) solutions were added to an equal volume of n-octanol (presaturated with 0.9% NaCl (w/v) ultrapure water). The heterogeneous mixtures were shaken vigorously for 30 min before centrifuging at 4000 rpm for 30 min to achieve phase separation. The final Pt concentration in the aqueous phase was determined again by ICP-MS. The logarithm of the ratio of Pt concentrations in the organic and aqueous phases was calculated to determine the log*P* values. All experiments were done in duplicate.

### Stability studies

Complexes **1A**, **1P**, **3A** and **3P** where dissolved in a minimum amount of DMF and diluted 1:20 with physiological saline (0.9% NaCl). Mass spectra were recorded at t = 0 and after 48 hours.

### Experiments with cultured human cells

The Pt(IV) compounds were dissolved in DMSO just before running the experiment and a calculated amount of drug solution was added to the cell growth medium to a final DMSO concentration of 0.5%, which had no detectable effect on cell viability. Cisplatin was dissolved in 0.9% NaCl solution.

### Cell cultures

Human breast (MCF-7), colon (HCT-15), lung (A549) and pancreatic (BxPC3) carcinoma cell lines together with melanoma (A375) cells were obtained from American Type Culture Collection (ATCC, Rockville, MD). The human ovarian cancer cell line 2008 and its cisplatin resistant variant, C13*, were kindly provided by Prof. G. Marverti (Department of Biomedical Science of Modena University, Italy). Human cervical carcinoma (A431) cells were kindly provided by Prof. F. Zunino (Division of Experimental Oncology B, Istituto Nazionale dei Tumori, Milan, Italy). Cell lines were maintained in the logarithmic phase at 37 °C in a 5% carbon dioxide atmosphere using the following culture media containing 10% fetal calf serum (Euroclone, Milan, Italy), antibiotics (50 units/mL penicillin and 50 μg/mL streptomycin) and 2 mM L-glutamine: (i) RPMI-1640 medium (Euroclone) for 2008, C13*, MCF-7, HCT-15, A431 and BxPC3 cells, (ii) F-12 HAM’S (Sigma Chemical Co.) for A549 cells, iii) DMEM (Sigma Chemical Co.) for A375 cells.

### Cytotoxicity assays

The growth inhibitory effect toward tumor cells was evaluated by means of the MTT assay. Briefly, (3–8) × 10^3^ cells/well, dependent upon the growth characteristics of the cell line, were seeded in 96-well microplates in growth medium (100 μL). After 24 h, the medium was removed and replaced with fresh medium containing the compound to be studied at the appropriate concentration. Triplicate cultures were established for each treatment. After 24, 48, or 72 h, each well was treated with 10 μL of a 5 mg/mL MTT saline solution and, after 5 h of incubation, 100 μL of a sodium dodecylsulfate (SDS) solution in 0.01 M HCl was added. After an overnight incubation, cell growth inhibition was detected by measuring the absorbance of each well at 570 nm using a Bio-Rad 680 microplate reader. The mean absorbance for each drug dose was expressed as a percentage of the control, untreated well absorbance and plotted vs drug concentration. The IC_50_ values, the drug concentrations that decrease the mean absorbance at 570 nm to 50% of that of untreated control wells, were calculated from the dose–response curves with the four-parameter logistic model (4PL). The final value is the mean ± S.D. of at least three independent experiments performed in triplicate.

### Cellular accumulation of Pt

A375 cells (2 × 10^6^) were seeded in 75 cm^2^ flasks in growth medium (20 mL). After 24 h, the medium was replaced and the cells incubated for 6 or 24 h with the tested complexes. Cell monolayers were washed twice with cold phosphate-buffered saline (PBS), harvested and counted. Samples were subjected to three freeze-thaw cycles at −80 °C, and then vigorously vortexed. The samples were treated with highly pure nitric acid (1 mL, Pt ≤ 0.01 mg·kg^−1^, Trace-SELECT Ultra, Sigma Chemical Co.) and transferred into a microwave teflon vessel. Subsequently, samples were submitted to standard mineralization procedures. Samples were analyzed for platinum by using a Varian AA Duo graphite furnace atomic absorption spectrometer (Varian, Palo Alto, CA, USA) at a wavelength of 324.7 nm. The calibration curve was obtained using known concentrations of standard solutions purchased from Sigma Chemical Co.

### DNA platination

A375 cells (3 × 10^6^) were seeded in 10 cm Petri dishes in 10 mL of culture medium. Subsequently, cells were treated with the tested complexes (5 μM) for 24 h. The DNA was extracted and purified by a commercial spin column quantification kit (Qiagen DNeasy Blood and Tissue Kit). Only highly purified samples (A260/A230 ≅ 1.8 and A280/A260 ≅ 2.0) were included for analysis to avoid any artifacts. The samples were completely dried and re-dissolved in 200 μL of Milli-Q water (18.2 MΩ) for at least 20 min at 65 °C in a shaking thermo-mixer, mineralized and analyzed for total Pt content by GF-AAS as described above.

### ROS production

The production of ROS was measured in A375 cells (10^4^ per well) grown for 24 h in 96-well plates in DMEM medium without phenol red (Sigma Chemical Co.). Cells were then washed with PBS and loaded with 10 μM 5-(and-6)-chloromethyl-2′,7′-dichlorodihydrofluorescein diacetate, acetyl ester (CM–H_2_DCFDA) (Molecular Probes-Invitrogen) for 25 min in the dark. Afterwards, cells were washed with PBS and incubated with increasing concentrations of the tested complexes. The fluorescence increase was estimated with a plate reader (Fluoroskan Ascent FL, Labsystem, Finland) at 485 (excitation) and 527 nm (emission). Antimycin (3 μM, Sigma Chemical Co.), a potent inhibitor of Complex III in the electron transport chain, was used as positive control.

### Mitochondrial membrane potential (ΔΨm)

The ΔΨm was assayed using the Mito-ID^®^ Membrane Potential Kit according to the manufacturer’s instructions (Enzo Life Sciences, Farmingdale, NY). Briefly, A375 cells (8 × 10^3^ per well) were seeded in 96-well plates, after 24 h, cells were washed with PBS and loaded with Mito-ID Detection Reagent for 30 min at 37 °C in the dark. Afterwards, cells were washed with PBS and incubated with increasing concentrations of the tested complexes. The fluorescence was estimated using a plate reader (Fluoroskan Ascent FL, Labsystem, Finland) at 490 (excitation) and 590 nm (emission).

### Hoechst 33258 staining

A375 cells were seeded into 8-well tissue-culture slides (BD Falcon, Bedford, MA, USA) at 5 × 10^4^ cells/well (0.8 cm^2^). After 24 h, cells were washed twice with PBS and following 48 and 72 h of treatment with IC_50_ doses of the tested compound, cells were stained for 5 min with 10 μg/mL of Hoechst 33258 (2′-(4-hydroxyphenyl)-5-(4-methyl-1-piperazinyl)-2,5′-bi-1H-benzimidazole trihydrochloride hydrate, Sigma–Aldrich) in PBS before being examined by fluorescence microscopy (Olympus).

### Caspase-3 activation

A375 cells (1 × 10^6^) were treated for 24 h with the IC_50_ doses of tested compounds, harvested and homogenized in a lysis buffer [1% Triton X-100, 320 nM sucrose, 5 mM EDTA, 10 mM Tris–HCl and 2 mM DTT (1,4-dithio-DL-threitol) buffer (pH 7.6)]. Protein aliquots (100 μg) were stained at 37 °C for 60 min with fluorescent caspase-3 substrate *N*-acetyl-Asp-Glu-Val-Asp-AMC (AMC = 7-amino-4-methylcoumarin) (Sigma Co.). Substrate hydrolysis was measured after 60 min by monitoring the release of AMC using a spectrofluorimeter (excitation at 370 nm, emission at 460 nm).

### Statistical analysis

All of the values are the means ± SD (standard deviation) of not less than three measurements starting from three different cell cultures. Multiple comparisons were made by ANOVA followed by the Tukey–Kramer multiple comparison test (**P < 0.01, *P < 0.05), using GraphPad Software.

## Additional Information

**How to cite this article**: Tolan, D. *et al*. Oxidative Stress Induced by Pt(IV) Pro-drugs Based on the Cisplatin Scaffold and Indole Carboxylic Acids in Axial Position. *Sci. Rep.* **6**, 29367; doi: 10.1038/srep29367 (2016).

## Supplementary Material

Supplementary Information

## Figures and Tables

**Figure 1:**
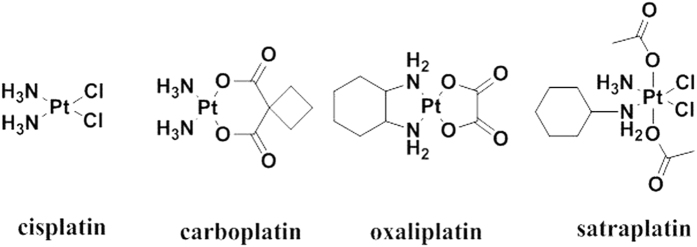
Chemical structures of the FDA-approved Pt(II) drugs and the Pt(IV) pro-drug in clinical trials, satraplatin.

**Figure 2:**
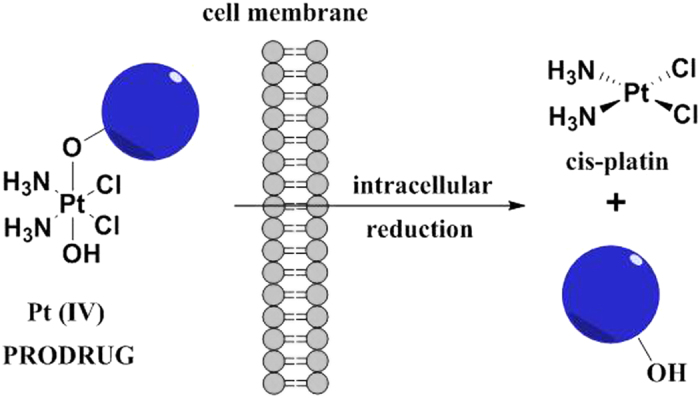
Activation by reduction of Pt(IV) complexes.

**Table 1:** IC_50_ values of the complexes in different cancer cell lines.

Complex	IC_50_(μM) ± S.D.					
	BxPC3	HCT-15	MCF-7	A431	A549	A375
1P	2.26 ± 0.99	3.66 ± 0.91	3.78 ± 0.16	1.62 ± 0.34	2.66 ± 0.54	0.76 ± 0.02
1A	2.64 ± 0.97	9.00 ± 3.13	3.62 ± 0.94	2.10 ± 1.04	3.62 ± 0.23	1.31 ± 0.82
2P	2.56 ± 0.65	14.00 ± 1.56	5.54 ± 0.67	3.00 ± 0.35	2.87 ± 1.59	1.35 ± 0.75
2A	3.32 ± 0.21	25.18 ± 0.89	3.70 ± 0.29	13.32 ± 0.84	3.75 ± 1.31	1.85 ± 0.23
3P	2.92 ± 1.13	5.94 ± 0.63	2.28 ± 0.46	2.00 ± 0.32	0.96 ± 0.06	1.51 ± 1.25
3A	2.56 ± 1.07	3.67 ± 1.31	3.76 ± 0.76	6.31 ± 0.94	2.59 ± 0.13	3.53 ± 0.60
4P	>50	41.99 ± 1.92	40.56 ± 0.08	29.25 ± 4.19	11.75 ± 1.06	8.66 ± 4.03
5P	19.50 ± 0.68	14.03 ± 0.14	21.01 ± 0.06	5.35 ± 2.11	3.62 ± 0.18	3.15 ± 0.02
4A	>50	>50	>50	3.94 ± 0.57	18.11 ± 4.32	48.52 ± 1.48
5A	44.44 ± 6.19	44.75 ± 0.07	43.26 ± 0.22	3.07 ± 0.31	2.80 ± 0.09	26.92 ± 4.04
IAA	>50	>50	>50	>50	>50	>50
IPA	>50	>50	>50	>50	>50	>50
*cisplatin*	10.17 ± 1.65	15.53 ± 2.48	7.60 ± 2.96	1.96 ± 0.84	12.64 ± 0.81	2.06 ± 1.01

Cells (3–8 × 10^3^ m^−1^) were treated for 72 h with increasing concentrations of tested compounds. Cytotoxicity was assessed by MTT test. IC_50_ values were calculated by four parameter logistic model (p < 0.05). S.D. = standard deviation.

**Table 2:** IC_50_ values of the compounds in cisplatin-sensitive and cisplatin-resistant cell lines.

IC_50_(μM) ± D.S.			
Complex	2008	C13*	RF
1P	1.45 ± 0.37	1.14 ± 0.50	0.8
1A	2.89 ± 1.22	2.51 ± 1.50	0.9
2P	8.37 ± 2.12	2.91 ± 0.64	0.4
2A	11.01 ± 2.14	3.44 ± 0.59	0.3
3A	9.97 ± 3.18	6.52 ± 1.96	0.7
3P	4.79 ± 0.69	1.47 ± 0.68	0.3
4P	>50	>50	−
5P	18.04 ± 1.33	21.85 ± 2.48	1.2
4A	>50	>50	−
5A	41.21 ± 0.75	46.58 ± 3.41	1.1
*cisplatin*	2.26 ± 1.06	23.73 ± ± 2.42	10.5

Cells (3–8 × 10^3^ m^−1^) were treated for 72 h with increasing concentrations of tested compounds. Cytotoxicity was assessed by MTT test. IC_50_ values were calculated by four parameter logistic model (p < 0.05). S.D. = standard deviation. Resistant Factor (RF) is defined as IC_50_ resistant/parent line.

**Table 3:** Log *P* (octanol/water) values of the Pt(IV) complexes.

Complex	Log *P*	Complex	Log *P*
1P	−0.110 ± 0.05	1A	−0.220 ± 0.04
2P	−0.051 ± 0.06	2A	−0.068 ± 0.07
3P	−0.028 ± 0.07	3A	−0.037 ± 0.04
4P	−0.100 ± 0.05	4A	−0.190 ± 0.06
5P	−0.080 ± 0.04	5A	−0.130 ± 0.05
