# Supplementary material for: Oxidative Stress Induced by Pt(IV) Pro-drugs Based on the Cisplatin Scaffold and Indole Carboxylic Acids in Axial Position
Source: Sci Rep. 2016 Jul 11;6:29367. doi: 10.1038/srep29367 (PMC4941645; doi:10.1038/srep29367)
Supplement: Supplementary Information [file srep29367-s1.pdf]

Oxidative Stress Induced by Pt(IV) Pro-drugs Based on the Cisplatin Scaffold and Indole Carboxylic Acids in Axial Position

Dina Tolan,[a,b] ‡ Valentina Gandin,[c] ‡ Liam Morrison,[d] Ahmed El-Nahas,[b] Cristina Marzano,[c] Diego Montagner,[a,e]* and Andrea Erxleben[a]*

[a] School of Chemistry, National University of Ireland, Galway, Ireland.
[b] Department of Chemistry, Faculty of Science, El-Menoufia University, Shebin El-Kom, Egypt.
[c] Department of Pharmaceutical and Pharmacological Sciences, University of Padua, Italy.
[d] Earth and Ocean Sciences, School of Natural Sciences and Ryan Institute, National University of Ireland, Galway, Ireland.
[e] Department of Chemistry, National University of Ireland, Maynooth, Ireland.

SUPPLEMENTARY INFORMATION

Synthetic Procedures and Characterization

Synthesis of the NHS esters of the carboxylic indole acetic acid (IAA) and indole propionic acid (IPA).

The carboxylic acid (5.3 mmol) and N-hydroxysuccinimide (0.6 g, 5.8 mmol) were dissolved in THF (10 mL). A solution of dicyclohexylcarbodiimide (1.2 g, 5.8 mmol) in THF (5 mL) was added dropwise. After stirring for 5 h at room temperature the precipitated dicyclohexylurea was removed by filtration. The filtrate was concentrated under reduced pressure and the residue was dissolved in ethyl acetate (30 mL) and left in the fridge overnight. The insoluble material formed was removed by filtration. The organic layer was washed with $NaHCO_3$ solution (4%; 60 mL) and with water (30 mL). After drying over Na_2SO_4, petroleum ether (50 mL) was added to the ethyl acetate solution. The resulting precipitate was collected by filtration, washed with petroleum ether and dried. (Yield 48 % for IAA ester and 50 % for IPA ester). ^{1}H NMR (400 MHz, $CDCl_3$): **IAA ester**: δ 8.15 (1H, NH), δ 7.60 (d, 1H, Ar-H), δ 7.32 (d, 1H, Ar-H), δ 7.25 (s, 1H, Ar-H), δ 7.19 (t, 1H, Ar-H), δ 7.14 (t, 1H, Ar-H), δ 4.1 (s, 2H, $-CH_2-$), δ 2.8 (s, 4H, $CO-CH_2-CH_2-CO$). **IPA ester**: δ 8.0 (1H, NH), δ 7.58 (d, 1H, Ar-H), δ 7.36 (d, 1H, Ar-H), δ 7.25 (s, 1H, Ar-H), δ 7.20 (t, 1H, Ar-H), δ 7.13 (t, 1H, Ar-H), δ 3.22 (t, 2H, $-CH_2-$), δ 3.01 (t, 2H, $-CH_2-$), δ 2.8 (s, 4H, $CO-CH_2-CH_2-CO$).

Synthesis of cis,cis,trans-[$Pt(NH_3)_2Cl_2(CO_2R)OH$] (1A and 1P)

The IAA-NHS ester (97.5 mg, 0.358 mmol) for complex **1A** and IPP –NHS ester (102 mg, 0.358 mmol) for complex **1P** were added to oxoplatin, cis,cis,trans-[$Pt(NH_3)_2Cl_2(OH)_2$], (150 mg, 0.449 mmol) in DMSO (10 mL). The reaction mixture was stirred at 50˚C for 20 h and then filtered to remove the unreacted oxoplatin. The solvent was evaporated using a freeze-dryer and the residue was dissolved in DMF (2 mL). The desired product was precipitated by adding diethyl ether. The solid was collected by centrifugation, washed several times with dichloromethane and diethyl ether to remove the residual DMF and finally dried under vacuum.

Complex 1A (orange). Yield: 92 mg (42%). ^{1}H NMR (400 MHz, DMSO-d_6) : δ 10.76 (s, 1H, NH), δ 7.50 (d, J = 8.0 Hz, 1 H, Ar-H), δ 7.27 (d, J = 8.0 Hz, 1 H, Ar-H), δ 7.23 (s, 1 H, Ar-H), δ 7.00 (t, J = 8.0 Hz, 1 H, Ar-H), δ 6.90 (t, J = 8.0 Hz, 1 H, Ar-H), δ 5.95 (br.

mult. $^{1}J_{NH}$ = 52.0 and $^{2}J_{PtH}$ = 51.5 Hz 6H, NH_3), δ 3.56 (s, 2 H, -CH_2-). ^{13}C NMR (100.61 MHz, DMSO-*d*$_6$): δ = 181.1 (C-1), 137.1(C-2), 128.7 (C-3), 124.8 (C-4), 121.8 (C-5), 120.0 (C-6), 119.1 (C-7), 112.1 (C-8), 110.5 (C-9), 35.0 (C-10) ppm. ^{195}Pt{^{1}H} NMR (107.6 MHz, DMF (D_2O)): δ 992 ppm. IR (cm^{-1}): 3388 m , 3201 s ($\nu_{N\text{-}H}$), 3063 s ($\nu_{C\text{-}H}$), 2916 w ($\nu_{C\text{-}H}$), 1646 s ($\nu_{C=O}$), 1617 s ($\nu_{C=C}$), 1457 m ($\nu_{C\text{-}C}$), 1414 m, 1338 s, 1312 s, 1252 m ($\nu_{C\text{-}C}$), 1221 s, 1098 m ($\delta_{C\text{-}H}$), 1025 m, 745 s ($\gamma_{C\text{-}H}$). ESI-MS (negative ion mode): *m/z* = 490.0 [M-H]$^-$. Anal. Calcd for $C_{10}H_{15}Cl_2N_3OPt$. C 24.45, H 3.08, N 8.55%. Found C, 24.85 H, 2.85 N. 8.30%.

Complex 1P (green). Yield: 100 mg (44%). ^{1}H NMR (400 MHz, DMSO-*d*$_6$) δ10.71 (s, 1H, NH), δ 7.43 (d, *J* = 7.8 Hz, 1H, Ar-H), 7.28 (d, *J* = 8.0 Hz, 1H, Ar-H), 7.10 (s, 1H, Ar-H), 7.01 (t, *J* = 8.0 Hz, 1H, Ar-H), δ 6.92 (t, *J* = 7.3 Hz, 1H, Ar-H), δ 5.95 (br. mult. $^{1}J_{NH}$ = 51.0 and $^{2}J_{PtH}$ = 52.5 Hz 6H, NH_3), δ 2.83 (t, 2H, -CH_2-), δ 2.49 (t, 2H, -CH_2-). ^{13}C NMR (100.61 MHz, DMSO-d6): δ = 181.1 (C-1), 136.7(C-2), 127.5 (C-3), 122.7 (C-4), 121.3 (C-5), 118.6(C6), 118.6 (C-7), 114.6 (C-8), 111.8 (C-9), 26.0 (C-10), 22.0 (C-11) ppm. ^{195}Pt {^{1}H} NMR (107.6 MHz, DMF (D_2O)): δ 987 ppm. IR (cm^{-1}): 3400 m, 3214 s ($\nu_{N\text{-}H}$), 3050 s ($\nu_{C\text{-}H}$), 2916 w ($\nu_{C\text{-}H}$), 1647 s ($\nu_{C=O}$), 1615 s ($\nu_{C=C}$), 1456 m ($\nu_{C\text{-}C}$), 1414 m, 1337 m, 1315 s, 1246 s ($\nu_{C\text{-}C}$), 1218 s, 1099 m ($\delta_{C\text{-}H}$), 1010 m, 743 s ($\gamma_{C\text{-}H}$) ESI-MS (negative ion mode): *m/z*= 504.01 [M-H]$^-$, 540.04. Anal. Calcd for $C_{11}H_{17}Cl_2N_3O_3Pt$. C 26.15, H 3.39, N 8.32%. Found C 26.68, H 3.78, N 8.12.

Synthesis of cis,cis,trans-[Pt(NH_3)$_2$$Cl_2$($RCO_2$)$_2$] (2A, 2P)

The IAA-NHS ester (2.43 g, 8.9 mmol) for complex **2A** and IPA-NHS ester (2.55 g, 8.9 mmol) for complex **2P** was added to cis,cis,trans-[Pt(NH_3)$_2$$Cl_2$($OH$)$_2$] (150 mg, 0.449 mmol) in DMSO (10 mL). The reaction mixture was stirred at 60˚C for 70 h. The solution was concentrated to 2 mL. The desired product was precipitated by adding water (30 mL) to the solution. The solid was collected by centrifugation, washed several times with dichloromethane and diethyl ether to remove the excess of the ligands, and finally dried under vacuum.

Complex 2A (brown). Yield: 96 mg (33%). ^{1}H NMR (400 MHz, DMSO-*d*$_6$): δ 10.81 (1H, NH), δ 7.52 (d, *J* = 7.8 Hz, 2 H, Ar-H), δ 7.28(d, *J* = 8.0 Hz, 2 H, Ar-H), δ 7.24 (s, 2 H, Ar-H), δ 7.01 (t, *J* = 8.0 Hz, 2 H, Ar-H), δ 6.91 (t, *J* = 8.8 Hz, 2 H, Ar-H), δ 6.57 (br, 6H, NH_3), δ 3.63 (s, 2 H, -CH_2-). ^{195}Pt{^{1}H} NMR (107.6 MHz, DMF (D_2O)): δ 1181 ppm. IR (cm^{-1}): 3384 m , 3251 m ($\nu_{N\text{-}H}$), 3063 m ($\nu_{C\text{-}H}$), 2916 m ($\nu_{C\text{-}H}$), 1641 s ($\nu_{C=O}$), 1618 s ($\nu_{C=C}$), 1457 m

($\nu_{C\text{-}C}$), 1419 m,1335 s, 1244 m ($\nu_{C\text{-}C}$), 1207 s, 1094 m ($\delta_{C\text{-}H}$), 1065 s, 1020 m, 743 s ($\gamma_{C\text{-}H}$). ESI-MS (negative ion mode): *m/z* = 683.04 $[M+Cl]^-$. Anal. Calcd for $C_{20}H_{22}Cl_2N_4O_4Pt$. C 37.05, H 3.42, N 8.64%. Found C 37.58, H 3.15, N 8.52%.

Complex 2P (yellow). Yield: 105 mg (35%). ^1H NMR (400 MHz, DMSO-*d*$_6$): δ 10.74 (1H, NH), δ 7.44 (d, *J* = 7.9 Hz, 2 H, Ar-H), δ 7.29 (d, *J* = 7.3 Hz, 2 H, Ar-H), δ 7.12 (s, 2 H, Ar-H), δ 7.02 (t, *J* = 8.28 Hz, 2 H, Ar-H), δ 6.94 (t, *J* = 6.9 Hz, 2 H, Ar-H), δ 6.56 (br, 6H, NH_3), δ 2.82 (t, 2 H, $-CH_2-$),), δ 2.59 (t, 2 H, $-CH_2-$). $^{195}Pt\{^1H\}$ NMR (107.6 MHz, DMF (D_2O)): δ 1178 ppm. IR (cm^{-1}): 3384 s , 3239 s ($\nu_{N\text{-}H}$), 3071 s ($\nu_{C\text{-}H}$), 2924 w ($\nu_{C\text{-}H}$), 1639 s ($\nu_{C=O}$), 1617 m ($\nu_{C=C}$), 1457 m ($\nu_{C\text{-}C}$), 1422 m, 1340 s, 1312 w, 1272 m ($\nu_{C\text{-}C}$), 1209 s, 1090 m ($\delta_{C\text{-}H}$), 1067 s, 1025 m, 744 s ($\gamma_{C\text{-}H}$). ESI-MS (negative ion mode): *m/z* = 711.06 $[M+Cl]^-$. Anal. Calcd for $C_{22}H_{26}Cl_2N_4O_4Pt$. C 39.06, H 3.87, N 8.28%. Found C 39.45, H 3.37, N 8.63%.

Synthesis of cis,cis,trans-[Pt(NH_3)$_2$$Cl_2$($RCO_2$)($C_6H_5CO_2$)] (3A, 3P) and cis,cis,trans-[Pt(NH_3)$_2$$Cl_2$($RCO_2$)($O_2CC_2H_4COOH$)] (4A, 4P).

Four equivalents of the corresponding anhydride and the mono-carboxylato complex were suspended in DMF. The reaction mixture was stirred overnight at 60˚C. The solution was then concentrated under reduced pressure. The desired product was precipitated by addition of diethyl ether. The solid was collected by centrifugation and washed with 4 mL of dichloromethane and diethyl ether to remove the residual DMF.

Complex 3A: Benzoic anhydride (184 mg, 0.816 mmol), 100 mg (0.201 mmol) of **1A** in DMF (6 mL); brownish red color. Yield: 70 mg (57%). ^1H NMR (400 MHz, DMSO-*d*$_6$): δ 10.83 (1H, NH), δ 7.85 (d, 2H, Ar-H), δ 7.55-7.37 (m, 4H, Ar-H), δ 7.26 (m, 1H, Ar-H), δ 7.17 (s, 1H, Ar-H), δ 7.02 (t, 1H, Ar-H), δ 6.92 (t, 1H, Ar-H), δ 6.65 (br, 6H, NH_3), δ 3.67 (s, 2H, $-CH_2-$).$^{195}Pt\{^1H\}$ NMR (107.6 MHz, DMF (D_2O)): δ 1170 ppm. IR (cm^{-1}): 3396 m, 3229 s ($\nu_{N\text{-}H}$), 3075 m ($\nu_{C\text{-}H}$), 2968 m ($\nu_{C\text{-}H}$), 1646 s ($\nu_{C=O}$), 1618 s ($\nu_{C=C}$), 1456 m ($\nu_{C\text{-}C}$), 1419 m, 1338 s, 1312 s, 1252 m ($\nu_{C\text{-}C}$), 1210 s, 1098 m ($\delta_{C\text{-}H}$), 1068 s, 747 s ($\gamma_{C\text{-}H}$). ESI-MS (negative ion mode): *m/z* = 630.01 $[M+Cl]^-$. Anal. Calcd for $C_{17}H_{19}Cl_2N_3O_4Pt$·DMF. C 35.94, H 3.92, N 8.38%. Found C 36.16, H 4.05, N 8.26%.

Complex 3P: Benzoic anhydride (178 mg, 0.796 mmol), 100 mg (0.198 mmol) of **1P** in DMF (6 mL); yellow color. Yield: 50 mg (41 %). ^1H NMR (400 MHz, DMSO-*d*$_6$): δ 10.75 (1H, NH), δ 7.85 (d, 2H, Ar-H), δ 7.51-7.38 (m, 4H, Ar-H), δ 7.29 (d, 1H, Ar-H), δ 7.14 (s,

1H, Ar-H), δ 7.03 (t, 1H, Ar-H), δ 6.94 (t, 1H, Ar-H), δ 6.56 (br, 6H, NH_3), δ 2.82 (t, 2H, -CH_2-), δ 2.62 (t, 2H, -CH_2-). $^{195}Pt\{^1H\}$ NMR (107.6 MHz, D_2O): δ 1166 ppm. IR (cm^{-1}): 3376 m, 3240 s (ν_{N-H}), 3190 s, 3055 m (ν_{C-H}), 2916 w (ν_{C-H}), 1649 s ($\nu_{C=O}$), 1630 s ($\nu_{C=C}$), 1449 m (ν_{C-C}), 1422 m, 1319 s, 1289 s, 1246 m (ν_{C-C}), 1213 m, 1132 s (δ_{C-H}), 1024 s, 749 s (γ_{C-H}). ESI-MS (negative ion mode): *m/z* = 644.03 $[M+Cl]^-$. Anal. Calcd for $C_{18}H_{21}Cl_2N_3O_4Pt$ ·DMF. C 36.96, H 4.14, N 8.21%. Found C 37.35, H 4.29, N 8.40%.

Complex 4A: Succinic anhydride (40.8 mg, 0.408 mmol), 50 mg (0.102 mmol) of **1A** in DMF (3 mL); brownish red color. Yield: 35 mg (55 %). ^1H NMR (400 MHz, DMSO-d_6): δ 12.05 (s, 1H, COOH), δ 10.82 (1H, NH), δ 7.51 (d, *J* = 8.4 Hz, 1H, Ar-H), δ 7.28 (d, *J* = 7.9 Hz, 1H, Ar-H), δ 7.24 (s, 1H, Ar-H), δ 7.01 (t, *J* = 8.0 Hz, 1H, Ar-H), δ 6.91 (t, *J* = 7.9 Hz, 1H, Ar-H), δ 6.51 (br, 6H, NH_3), δ 3.63 (s, 2H, -CH_2-) δ 2.39-2.49 (m, 4H, $COCH_2CH_2CO$). $^{195}Pt\{^1H\}$ NMR (107.6 MHz, DMF (D_2O)): δ 1182 ppm. IR (cm^{-1}): 3388 m, 3192 s (ν_{N-H}), 3083 m (ν_{C-H}), 2920 w (ν_{C-H}), 1706 s ($\nu_{C=O}$), 1648 s ($\nu_{C=O}$), 1617 w ($\nu_{C=C}$), 1583 w, 1458 m (ν_{C-C}), 1415 m, 1335 s, 1300 s, 1248 m (ν_{C-C}), 1216 m, 1169 s (δ_{C-H}), 1024 m, 746 s (γ_{C-H}). ESI-MS (negative ion mode): *m/z* = 590.02 $[M-H]^-$. Anal. Calcd for $C_{14}H_{19}Cl_2N_3O_6Pt$. C 28.44, H 3.24, N 7.11%. Found C 28.89, H 3.61, N 7.50%.

Complex 4P: Succinic anhydride (39.6 mg, 0.396 mmol), 50 mg (0.099 mmol) of **1P** in DMF (3 mL); brown color. Yield: 30 mg (47 %). ^1H NMR (400 MHz, DMSO-d_6): δ 12.04 (s, 1H, COOH), δ 10.74 (1H, NH), δ 7.43 (d, *J* = 7.8 Hz, 1H, Ar-H), δ 7.28 (d, *J* = 8.1 Hz, 1H, Ar-H), δ 7.11 (s, 1H, Ar-H), δ 7.02 (t, *J* = 7.9 Hz, 1H, Ar-H), δ 6.93 (t, *J* = 7.5 Hz, 1H, Ar-H), δ 6.51 (br, 6H, NH_3), δ 2.83 (t, 2H, -CH_2-), δ 2.56 (t, 2H, -CH_2-), δ 2.39-2.49 (m, 4H, $COCH_2CH_2CO$). $^{195}Pt\{^1H\}$ NMR (107.6 MHz, DMF (D_2O)): δ 1180 ppm. IR (cm^{-1}): 3396 m, 3186 s (ν_{N-H}), 3070 m (ν_{C-H}), 2916 w (ν_{C-H}), 1712 s ($\nu_{C=O}$), 1642 s ($\nu_{C=O}$), 1625 w ($\nu_{C=C}$), 1575 w, 1454 m (ν_{C-C}), 1415 m, 1310 s, 1292 s, 1240 m (ν_{C-C}), 1212 m, 1169 s (δ_{C-H}), 1010 m, 746 s (γ_{C-H}). ESI-MS (negative ion mode): *m/z* = 604.03 $[M-H]^-$, 640.00. Anal. Calcd for $C_{15}H_{21}Cl_2N_3O_6Pt$. C 29.76, H 3.50, N 6.94%. Found C 29.87, H 3.81, N 6.65%.

General procedure for the synthesis of cis,cis,trans-$[Pt(NH_3)_2Cl_2(RCO_2R)(CH_3CO_2)]$ (5A, 5P)

The corresponding mono–carboxylato complex **1A** or **1P** (50 mg) was stirred at room temperature in acetic anhydride (5 mL) for 20 h. The reaction mixture was lyophilized

and washed with diethyl ether (2 x 5 mL) to yield complex **5A** as a brown-red precipitate and complex **5P** as a brown precipitate.

Complex 5A: yield 29 mg (53%): ^{1}H NMR (400 MHz, DMSO-d$_6$) : δ 10.81 (1H, NH), δ 7.51 (d, *J* = 7.66 Hz, 1H, Ar-H), δ 7.28(d, *J* = 8.0 Hz, 1H, Ar-H), δ 7.24 (s, 1H, Ar-H), δ 7.01 (t, *J* = 8.0 Hz, 1H, Ar-H), δ 6.91 (t, *J* = 7.84 Hz, 1H, Ar-H), δ 6.54 (br, 6H, NH_3), δ 3.62 (s, 2H, -CH_2-), δ 1.91 (s, 3H, CH_3COO). ^{195}Pt{^{1}H} NMR (107.6 MHz, DMF (D_2O)): δ 1182 ppm. IR (cm^{-1}): 3396 w, 3216 s (ν_{N-H}), 3075 m (ν_{C-H}), 2916 w (ν_{C-H}), 1650 s ($\nu_{C=O}$), 1619 s ($\nu_{C=C}$), 1458 m (ν_{C-C}), 1415 m, 1357 m, 1289 s, 1219 m (ν_{C-C}), 1135 s (δ_{C-H}), 1016 s, 749 s (γ_{C-H}). ESI-MS (negative ion mode): *m/z* = 532.01 [M-H]$^-$, 567.0. Anal. Calcd for $C_{12}H_{17}Cl_2N_3O_4Pt$. C 27.03, H 3.21, N 7.88%. Found C 26.75, H 3.53, N 7.62%.

Complex 5P: yield 27 mg (50%): ^{1}H NMR (400 MHz, DMSO-d$_6$): δ 10.74 (1H, NH), δ 7.41 (d, *J* = 8.2 Hz ,1H, Ar-H), δ 7.28(d, *J* = 8.0 Hz, 1H, Ar-H), δ 7.12 (s, 1H, Ar-H), δ 7.02 (t, *J* = 7.12 Hz, 1H, Ar-H), δ 6.93 (t, *J* = 8.72 Hz, 1H, Ar-H), δ 6.53 (br, 6H, NH_3), δ 2.90 (t, 2H, -CH_2-), δ 2.62 (t, 2H, -CH_2-), δ 1.88 (s, 3H, CH_3COO). ^{195}Pt{^{1}H} NMR (107.6 MHz, DMF (D_2O)): δ 1179 ppm. IR (cm^{-1}): 3384 w, 3204 s (ν_{N-H}), 3079 m (ν_{C-H}), 2916 w (ν_{C-H}), 1650 m ($\nu_{C=O}$), 1622 s ($\nu_{C=C}$), 1457 m (ν_{C-C}), 1423 m, 1359 m, 1292 s, 1220 m (ν_{C-C}), 1123 m (δ_{C-H}), 1021 s, 745 s (γ_{C-H}). ESI-MS (negative ion mode): *m/z* = 546.03 [M-H]$^-$. Anal. Calcd for $C_{13}H_{19}Cl_2N_3O_4Pt$. C 28.53, H 3.50, N 7.68 Found C 28.94, H 3.13, N 7.58.

Spectra of 1A

^{1}H NMR (DMSO-d_6)

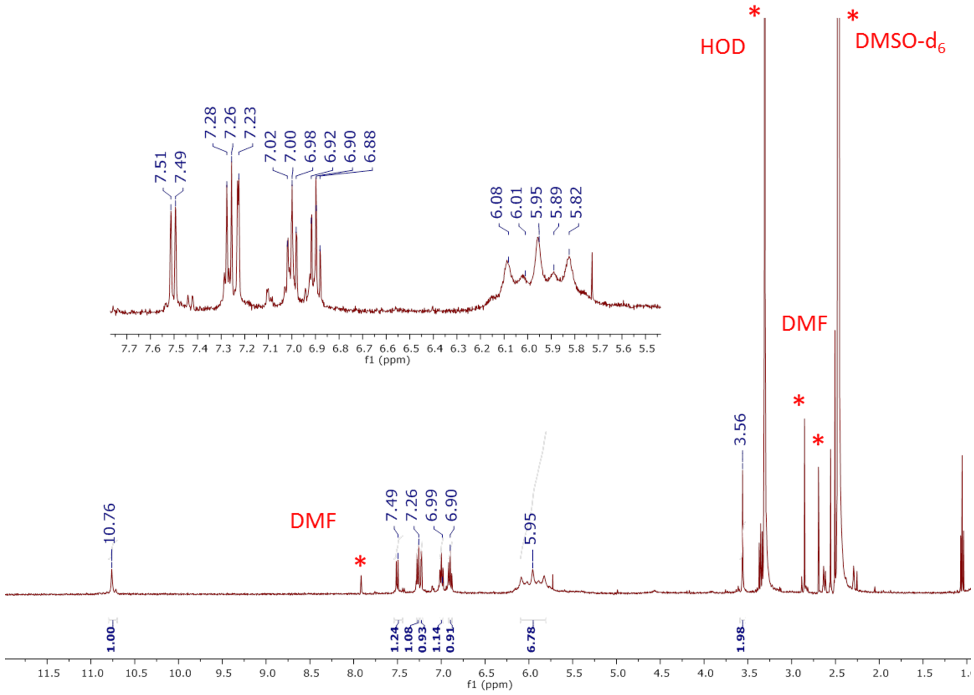

$^{13}C\{^{1}H\}$ NMR (DMSO-d_6)

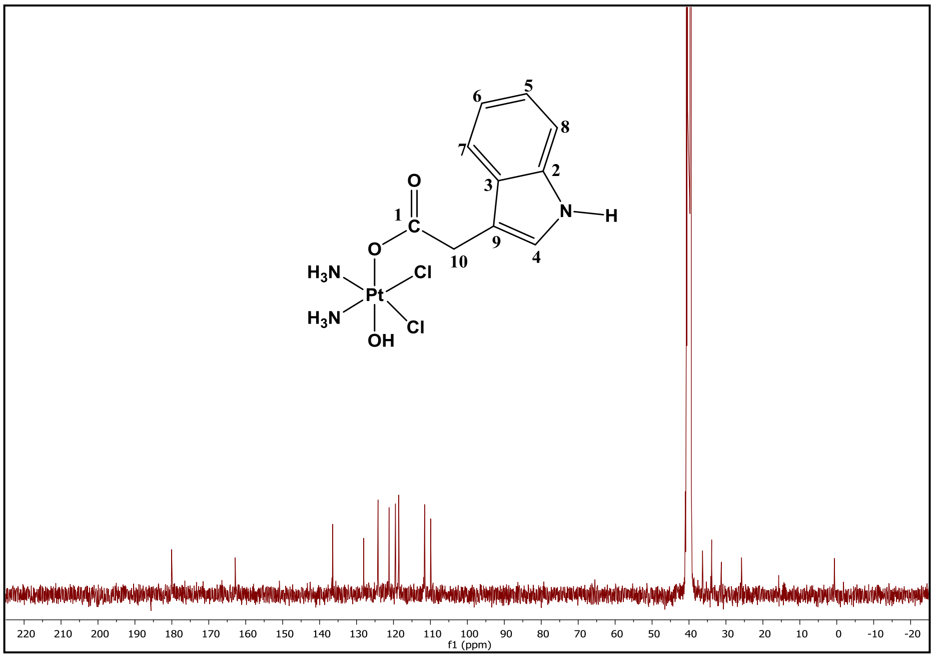

$^{195}Pt\{^{1}H\}$ NMR DMF (D_2O)

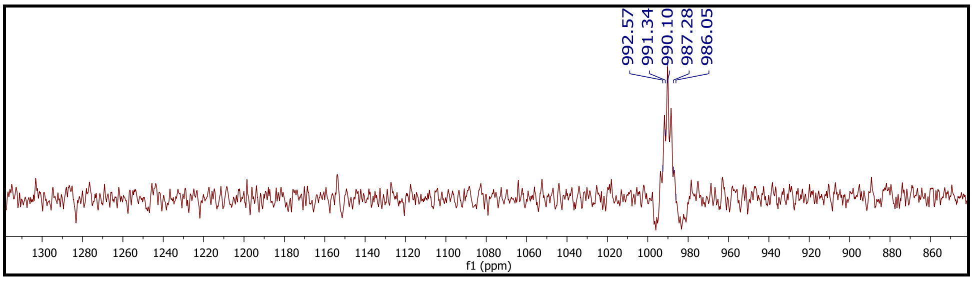

ESI-MS (negative mode)

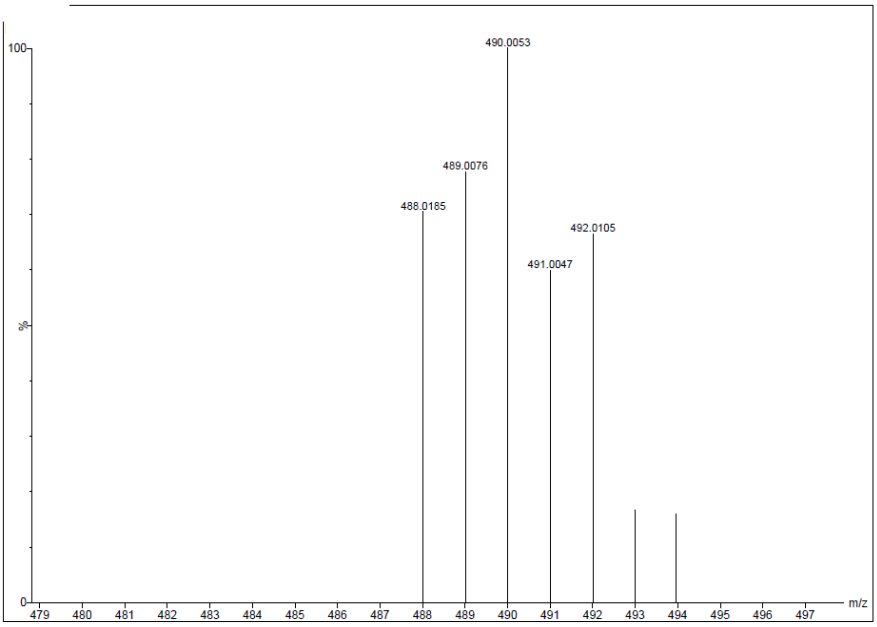

Spectra of 2A

^{1}H NMR (DMSO-d_6)

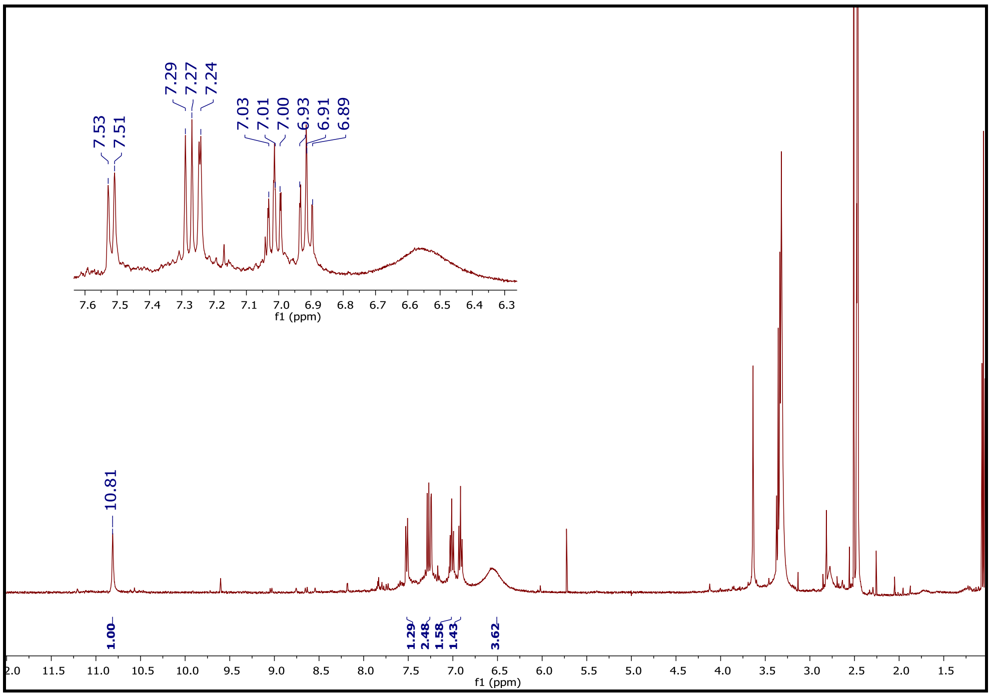

Peaks at δ 3.33 and 2.50 are due to H_2O and DMSO, respectively

^{195}Pt{^{1}H} NMR DMF(D_2O)

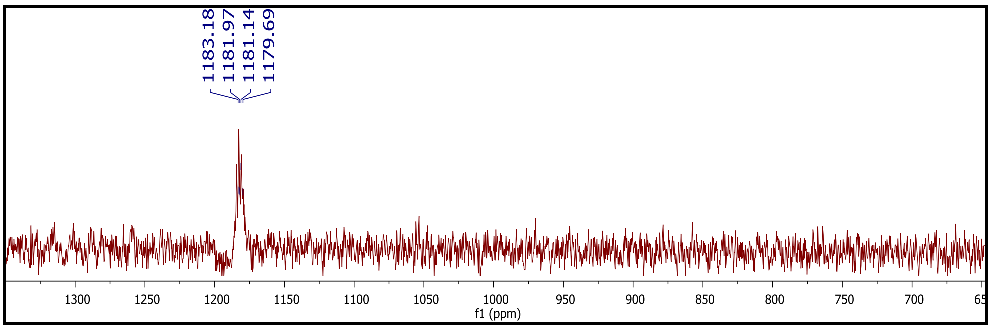

ESI-MS (negative mode)

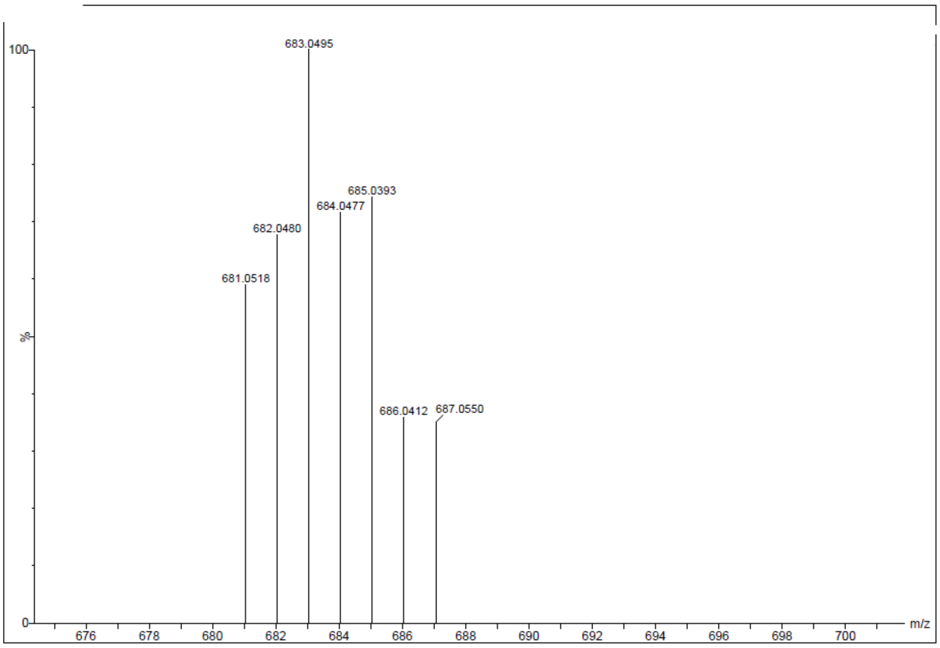

Spectra of 3A

^{1}H NMR (DMSO-d_6)

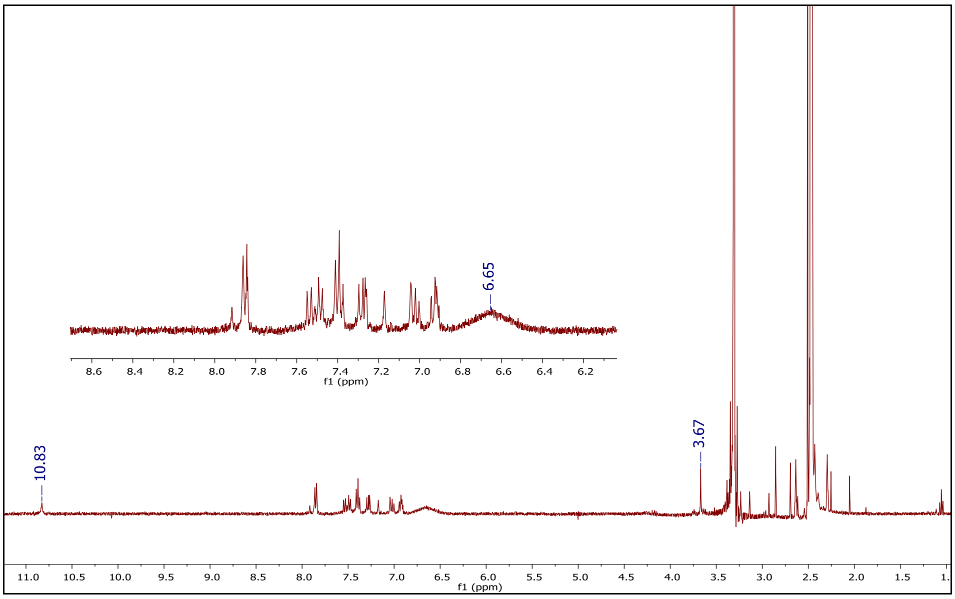

Peaks at δ 3.33 and 2.50 are due to H_2O and DMSO, respectively

$^{195}Pt\{^{1}H\}$ NMR DMF(D_2O)

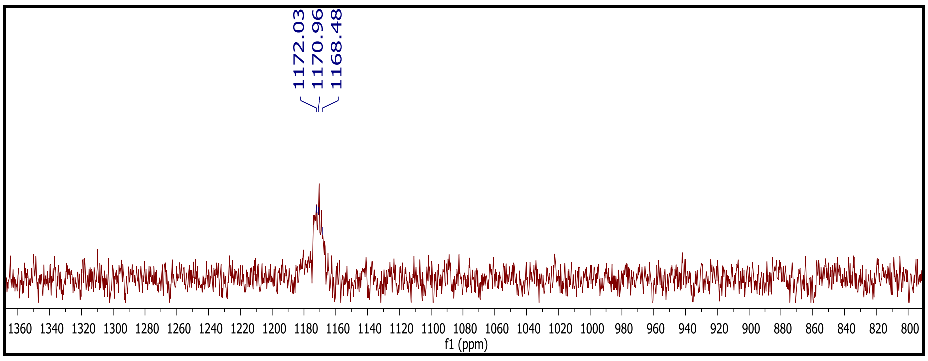

ESI-MS (negative mode)

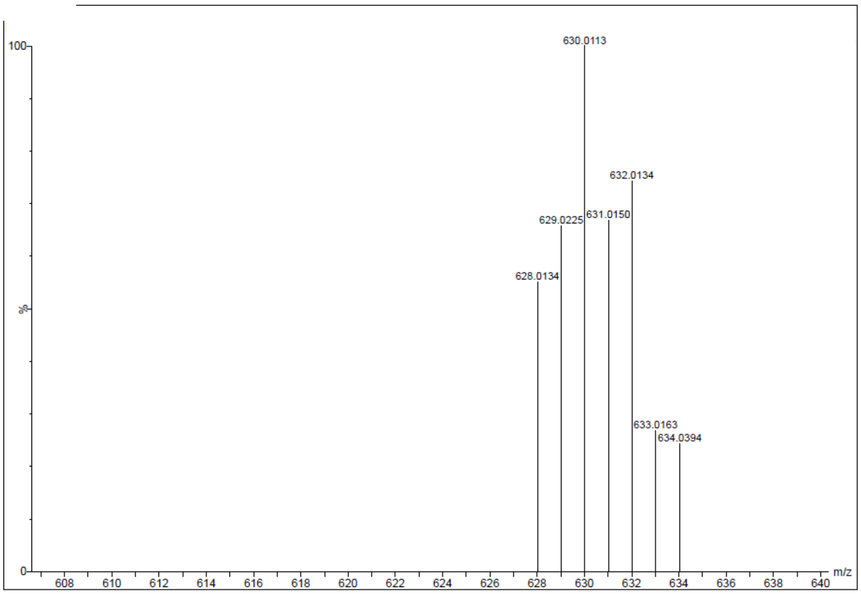

Spectra of 4A

^{1}H NMR (DMSO-d_6)

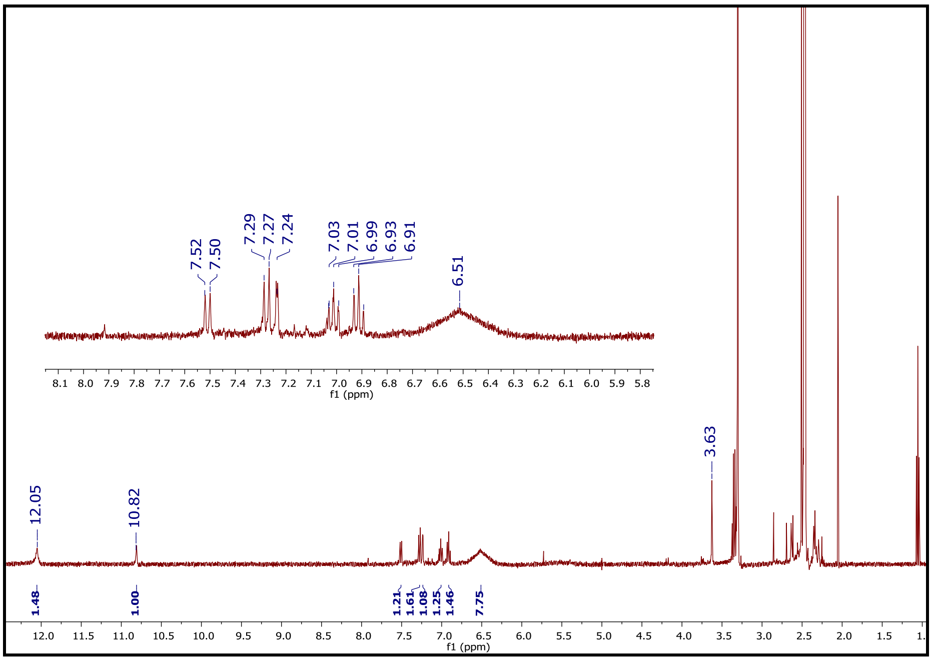

Peaks at δ 3.33 and 2.50 are due to H_2O and DMSO, respectively

$^{195}Pt\{^{1}H\}$ NMR DMF(D_2O)

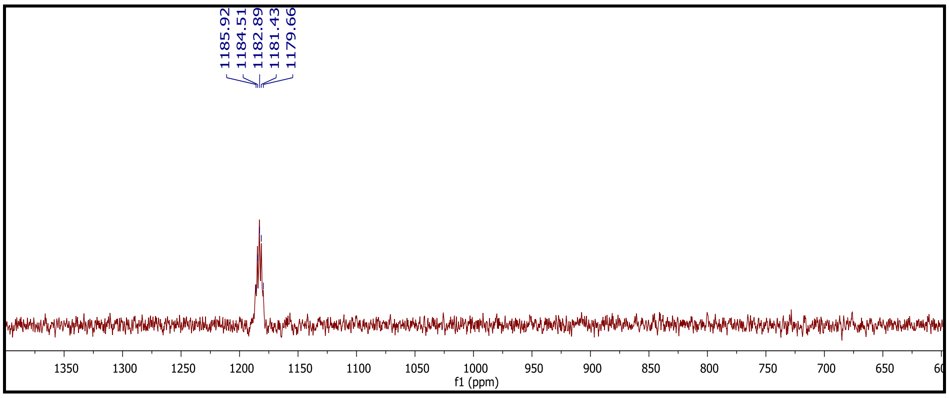

ESI-MS (negative mode)

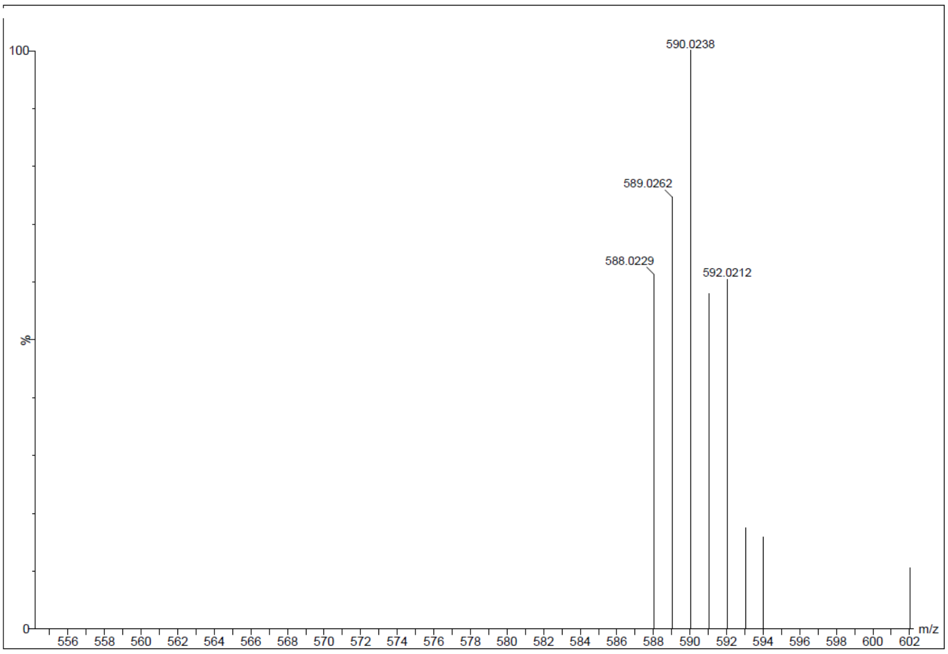

Spectra of 5A

^{1}H NMR (DMSO-d_6)

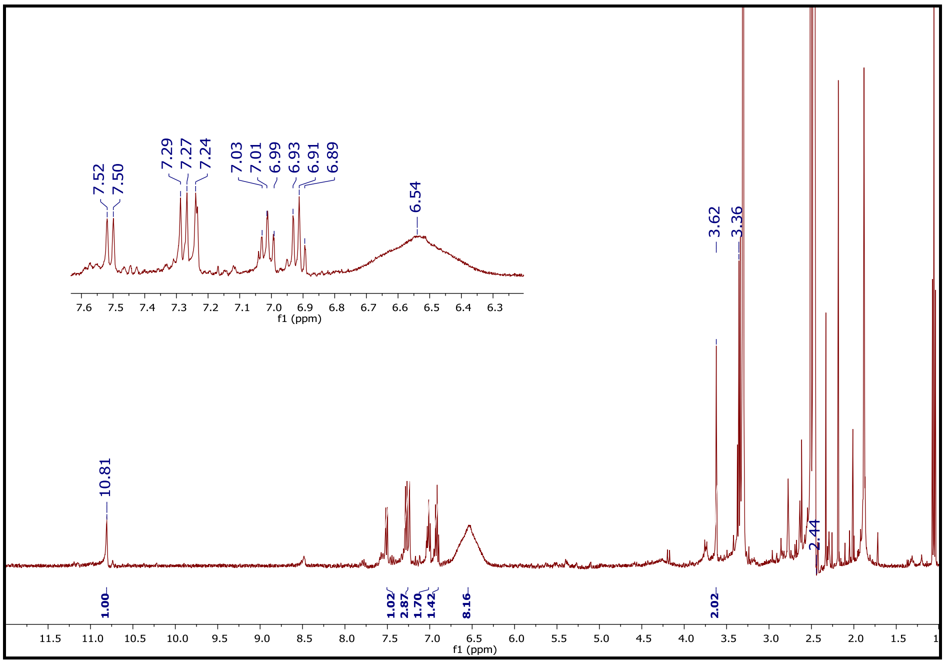

Peaks at δ 3.33 and 2.50 are due to H_2O and DMSO, respectively

$^{195}Pt\{^1H\}$ NMR DMF(D_2O)

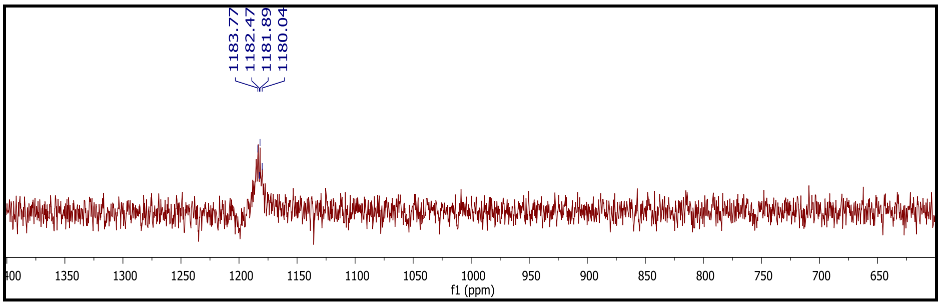

ESI-MS (negative mode)

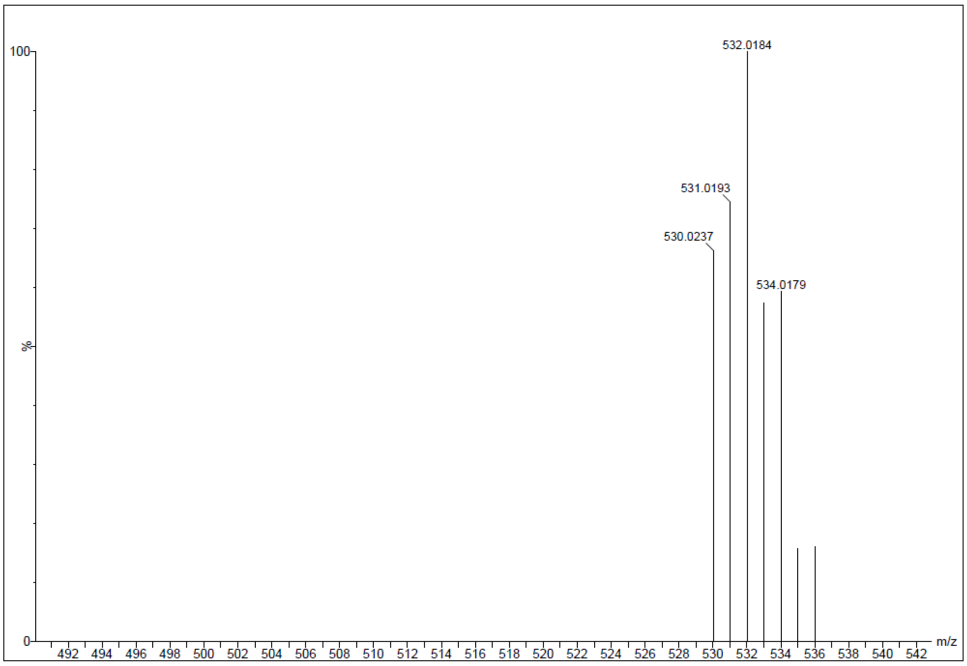

Spectra of 1P

^{1}H NMR (DMSO-d_6)

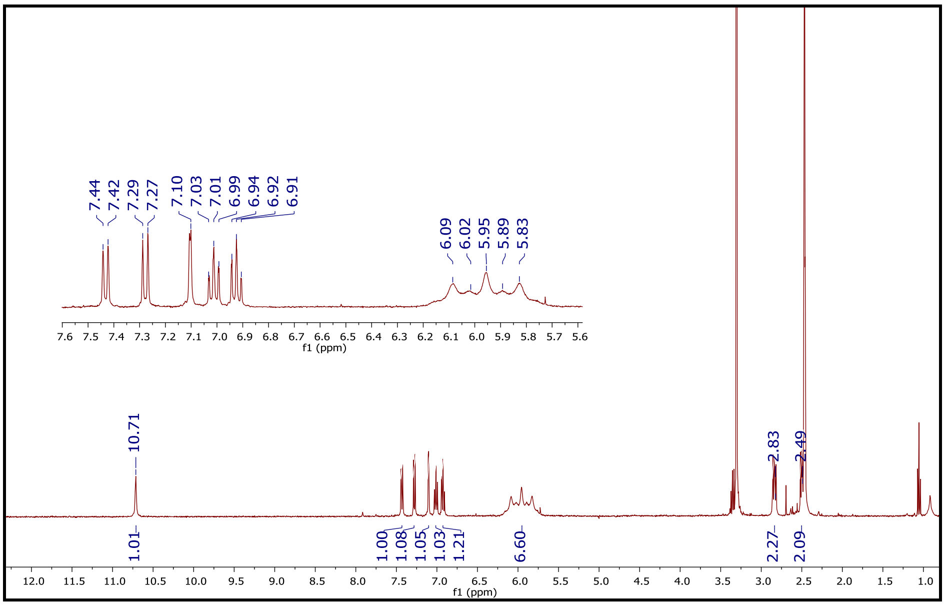

Peaks at δ 3.33 and 2.50 are due to H_2O and DMSO, respectively

^{13}C{^{1}H} NMR (DMSO-d_6)

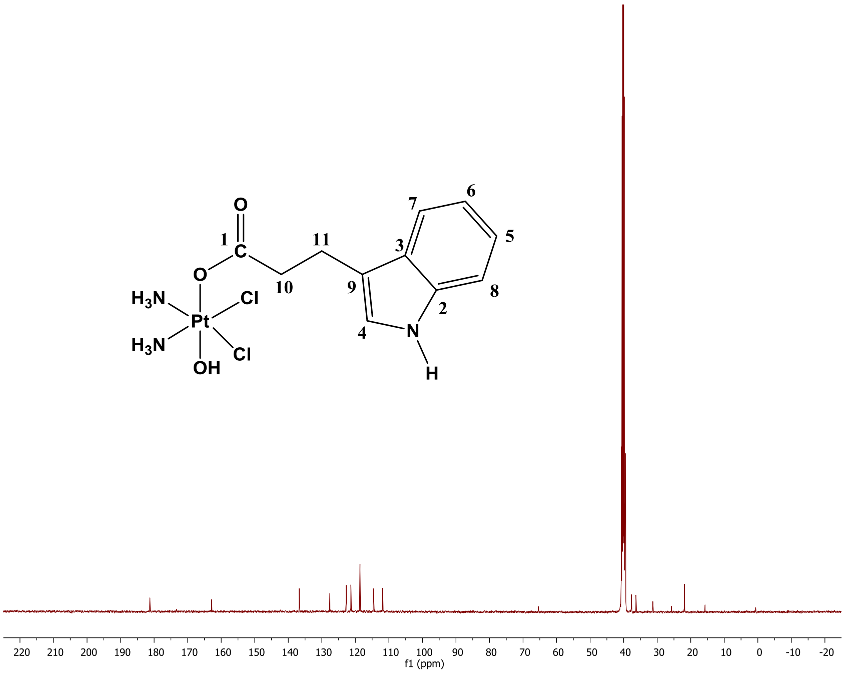

$^{195}Pt\{^{1}H\}$ *NMR DMF(D_2O)*

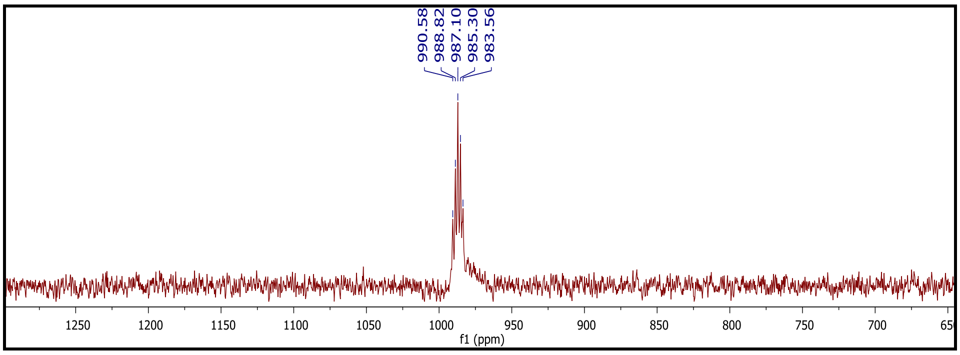

ESI-MS (negative mode)

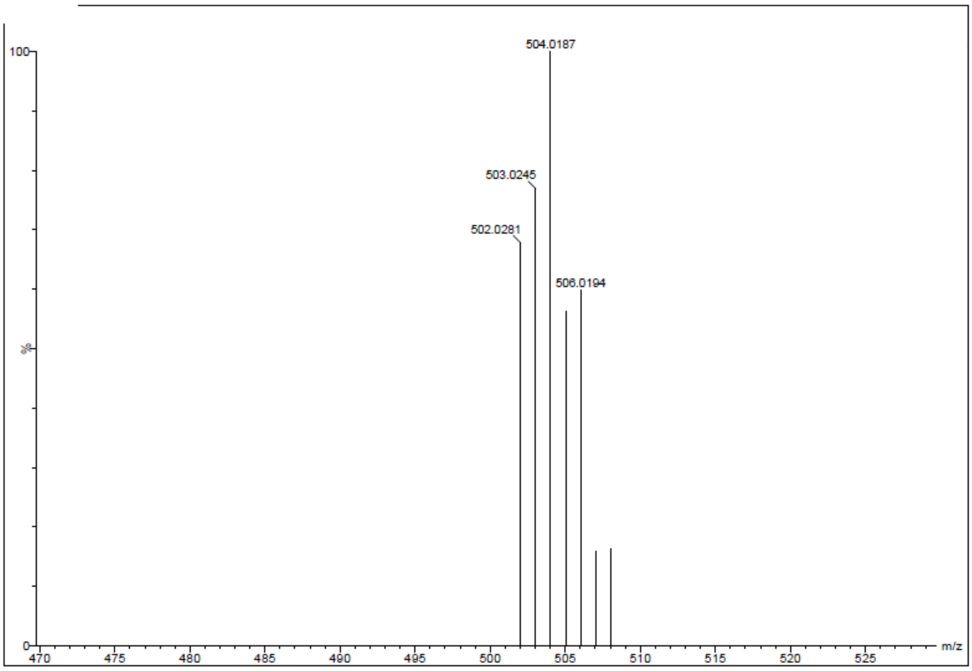

Spectra of 2P

^{1}H NMR (DMSO-d_6)

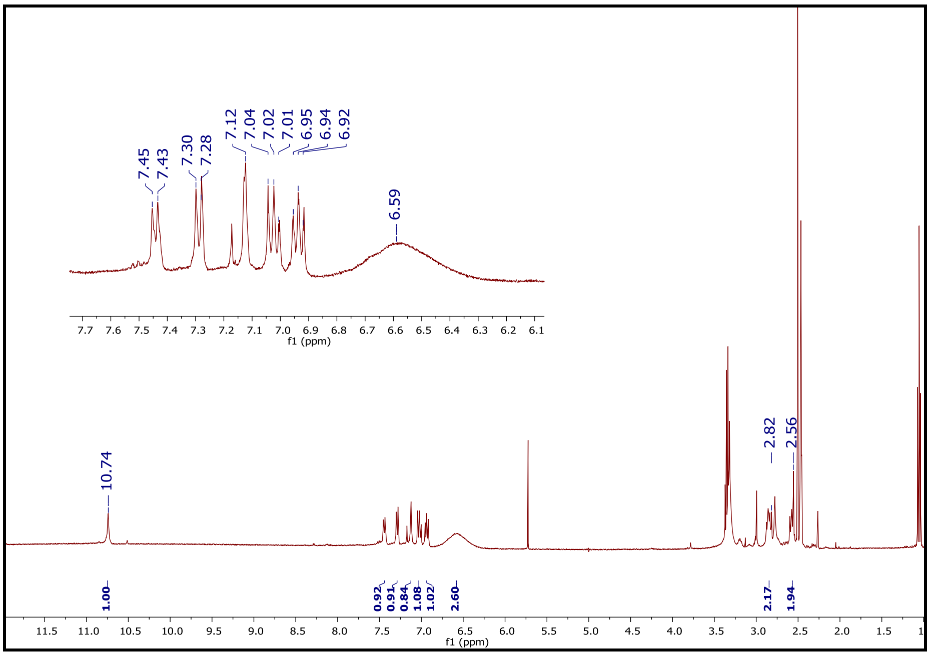

Peaks at δ 3.33 and 2.50 are due to H_2O and DMSO, respectively

^{195}Pt{^{1}H} NMR DMF(D_2O)

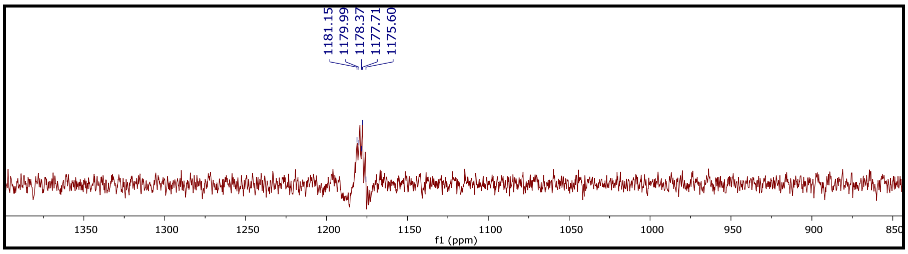

ESI-MS (negative mode)

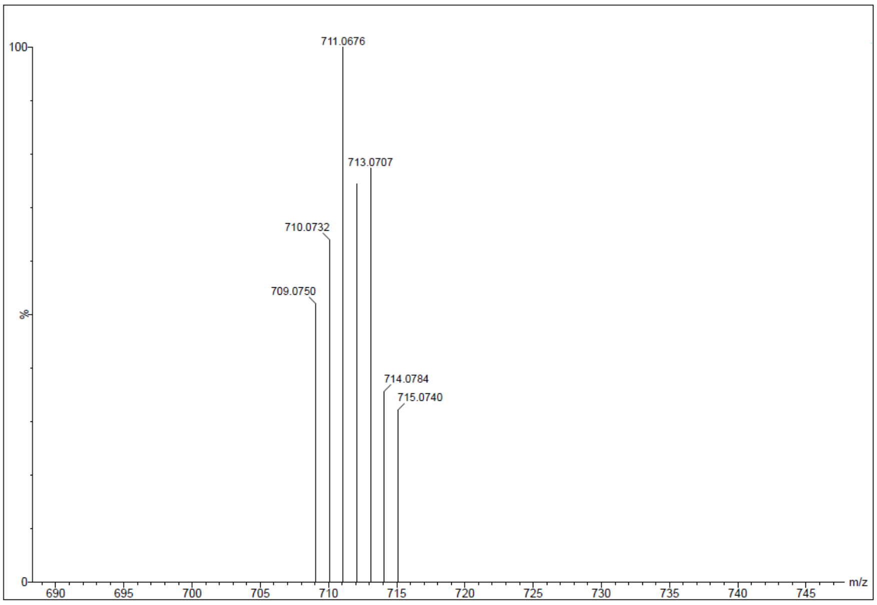

Spectra of 3P

^{1}H NMR (DMSO-d_6)

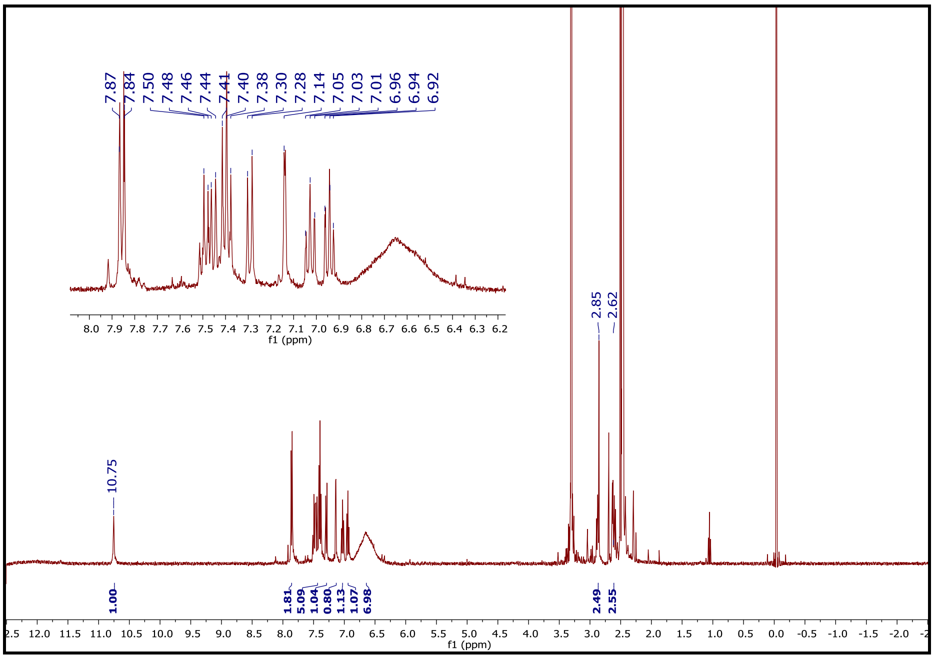

Peaks at δ 3.33 and 2.50 are due to H_2O and DMSO, respectively

$^{195}Pt\{^1H\}$ NMR DMF(D_2O)

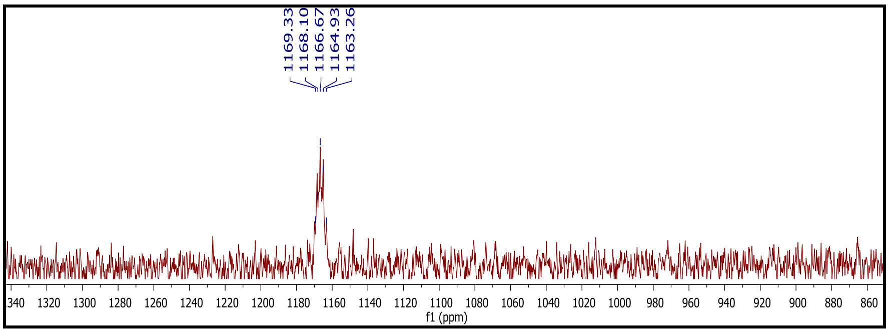

ESI-MS (negative mode)

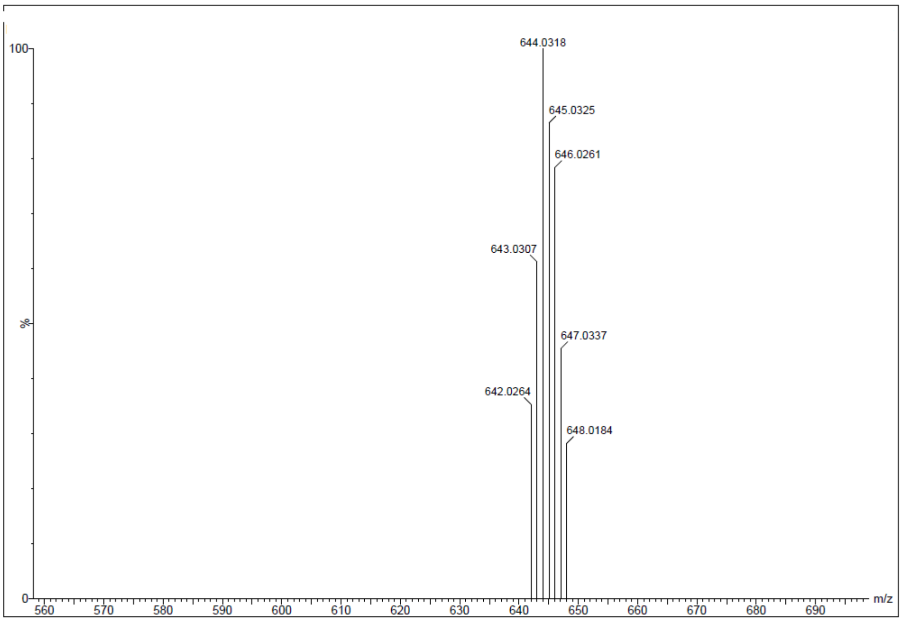

Spectra of 4P

^{1}H NMR (DMSO-d$_6$)

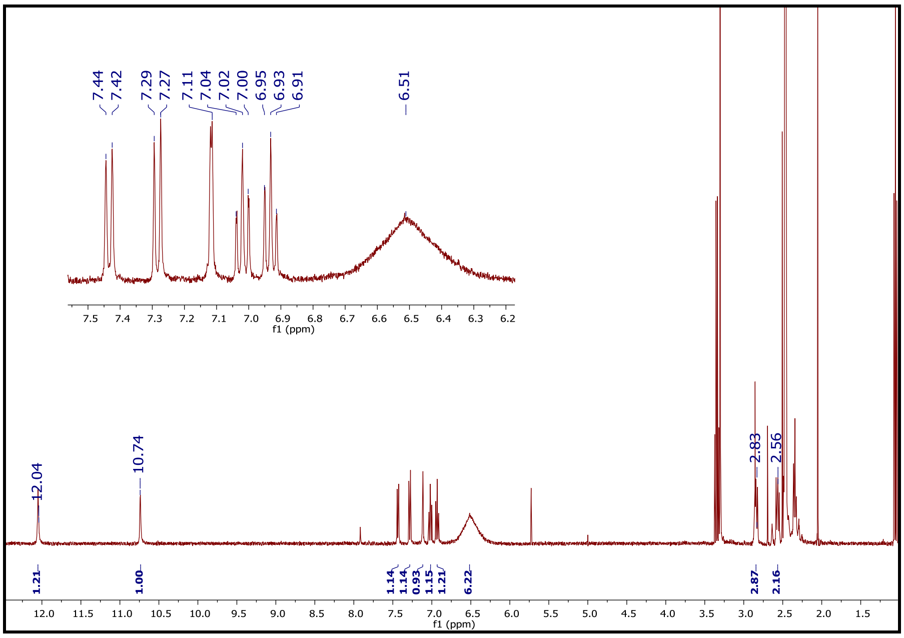

Peaks at δ 3.33 and 2.50 are due to H_2O and DMSO, respectively

^{195}Pt{^{1}H} NMR DMF(D$_2$O)

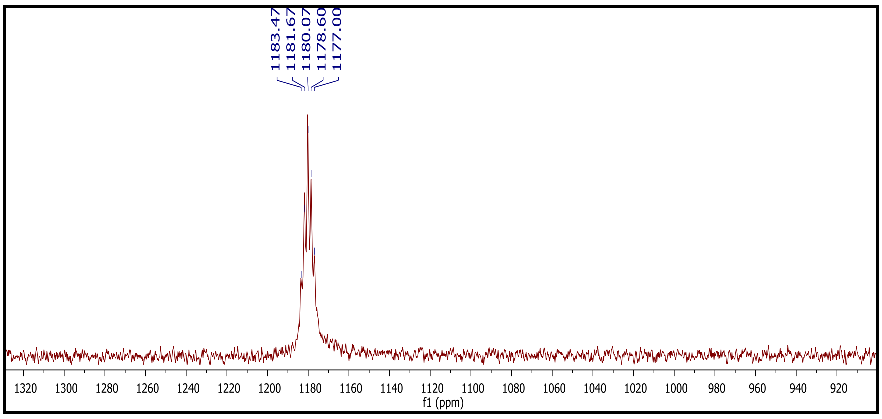

ESI-MS (negative mode)

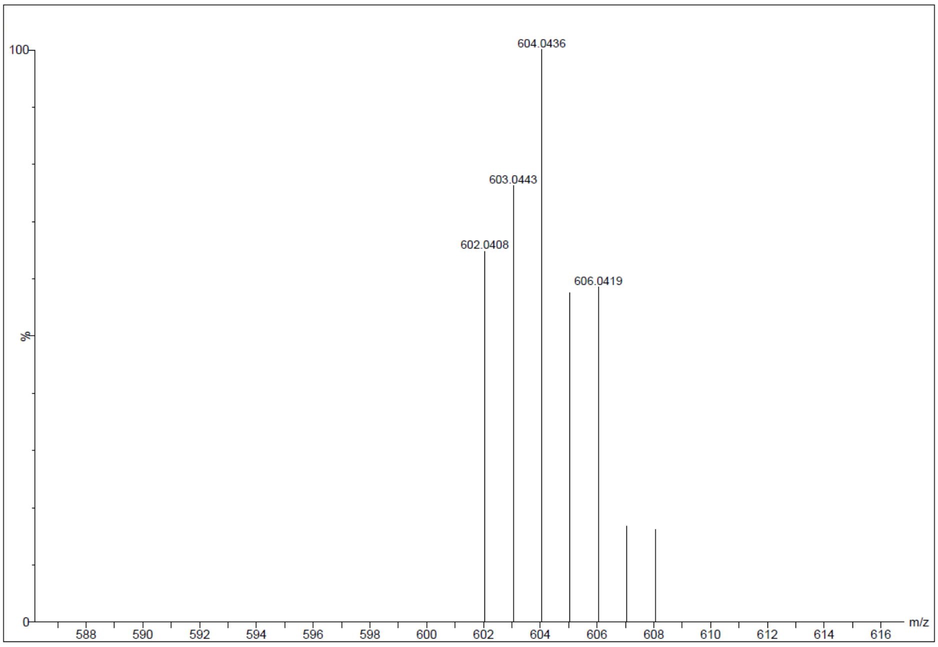

Spectra of 5P

^{1}H NMR (DMSO-d_6)

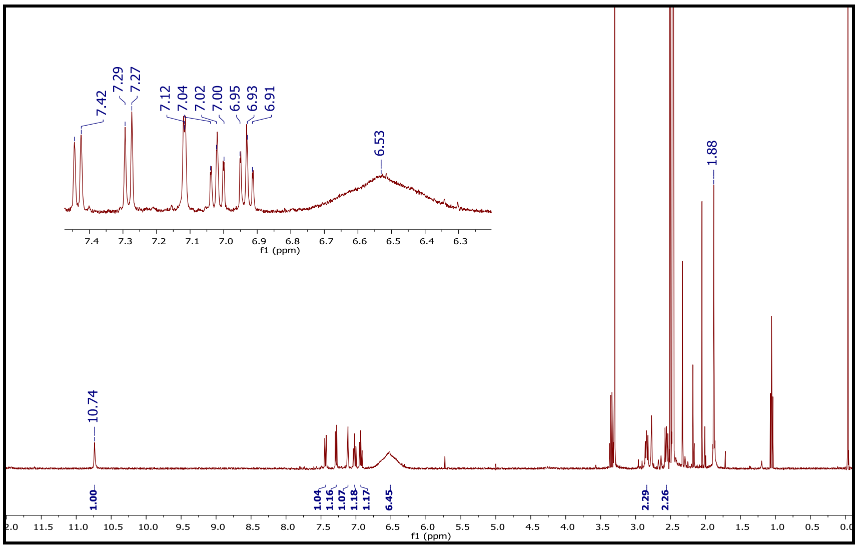

Peaks at δ 3.33 and 2.50 are due to H_2O and DMSO, respectively

$^{195}Pt\{^{1}H\}$ *NMR DMF(D_2O)*

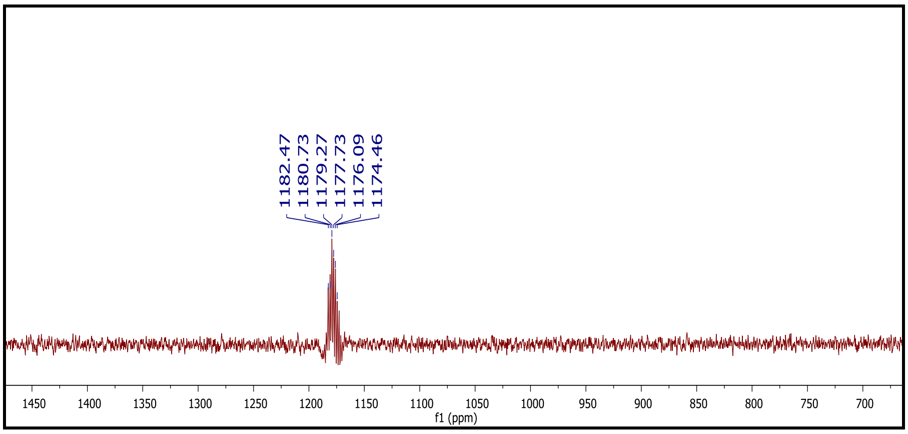

ESI-MS (negative mode)

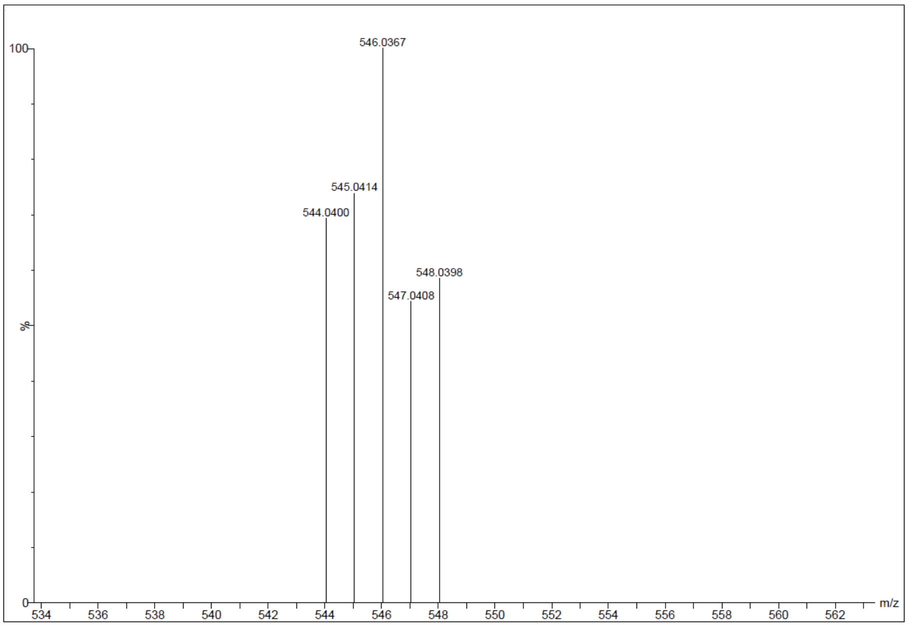

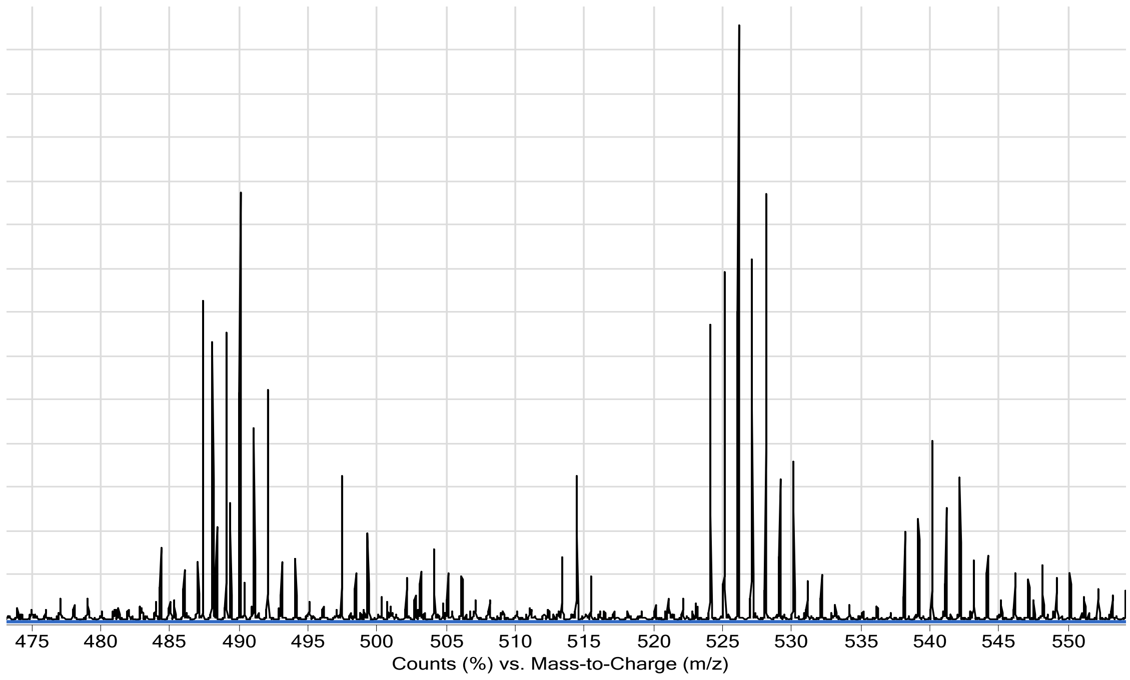

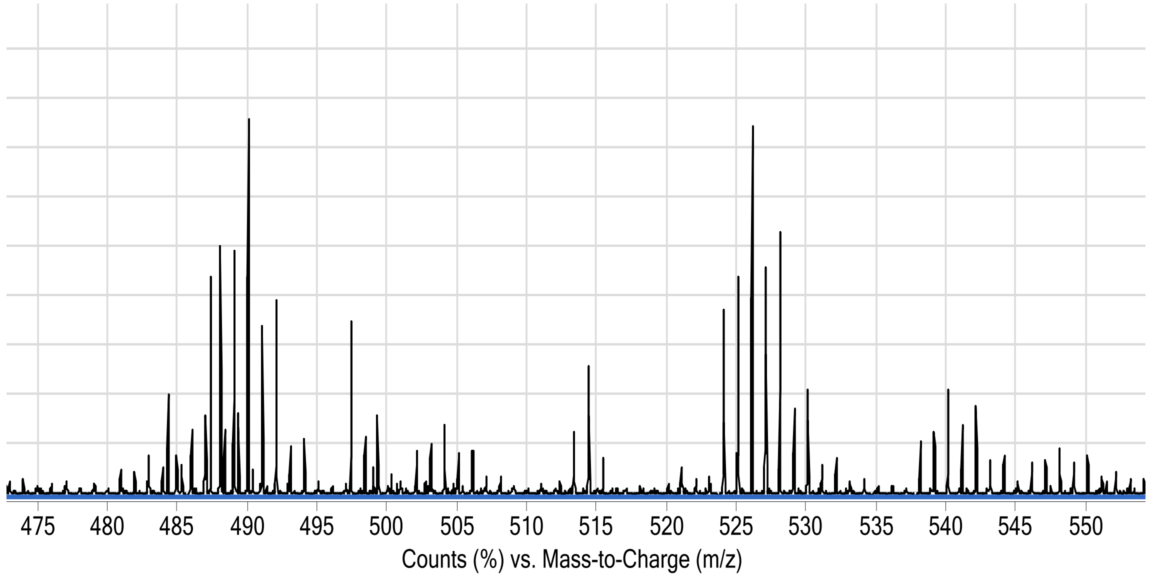

Mass spectrum of **1A** in physiological solution (0.9 % NaCl): top after dissolution; bottom after 48 h.

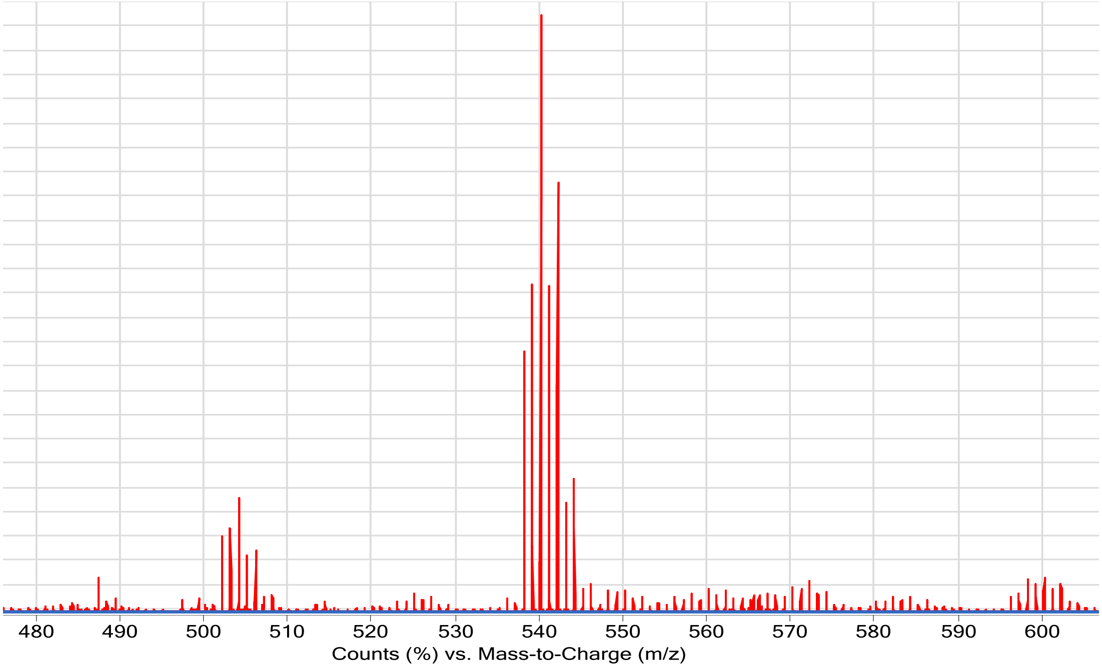

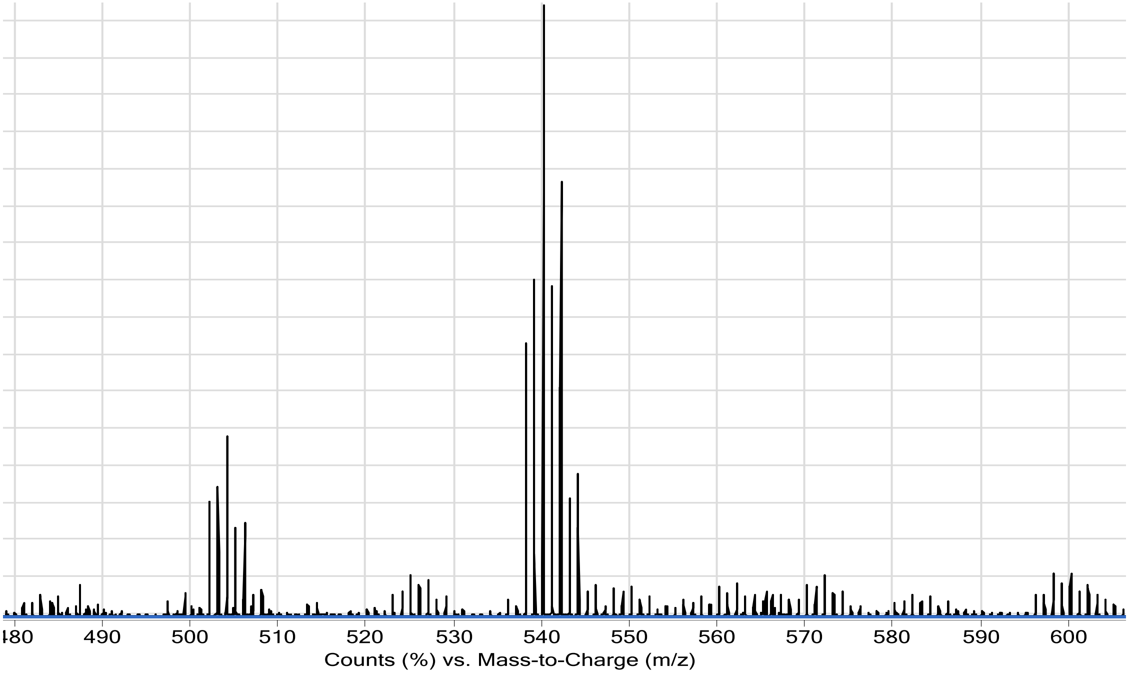

Mass spectrum of **1P** in physiological solution (0.9 % NaCl): top after dissolution; bottom after 48 h.

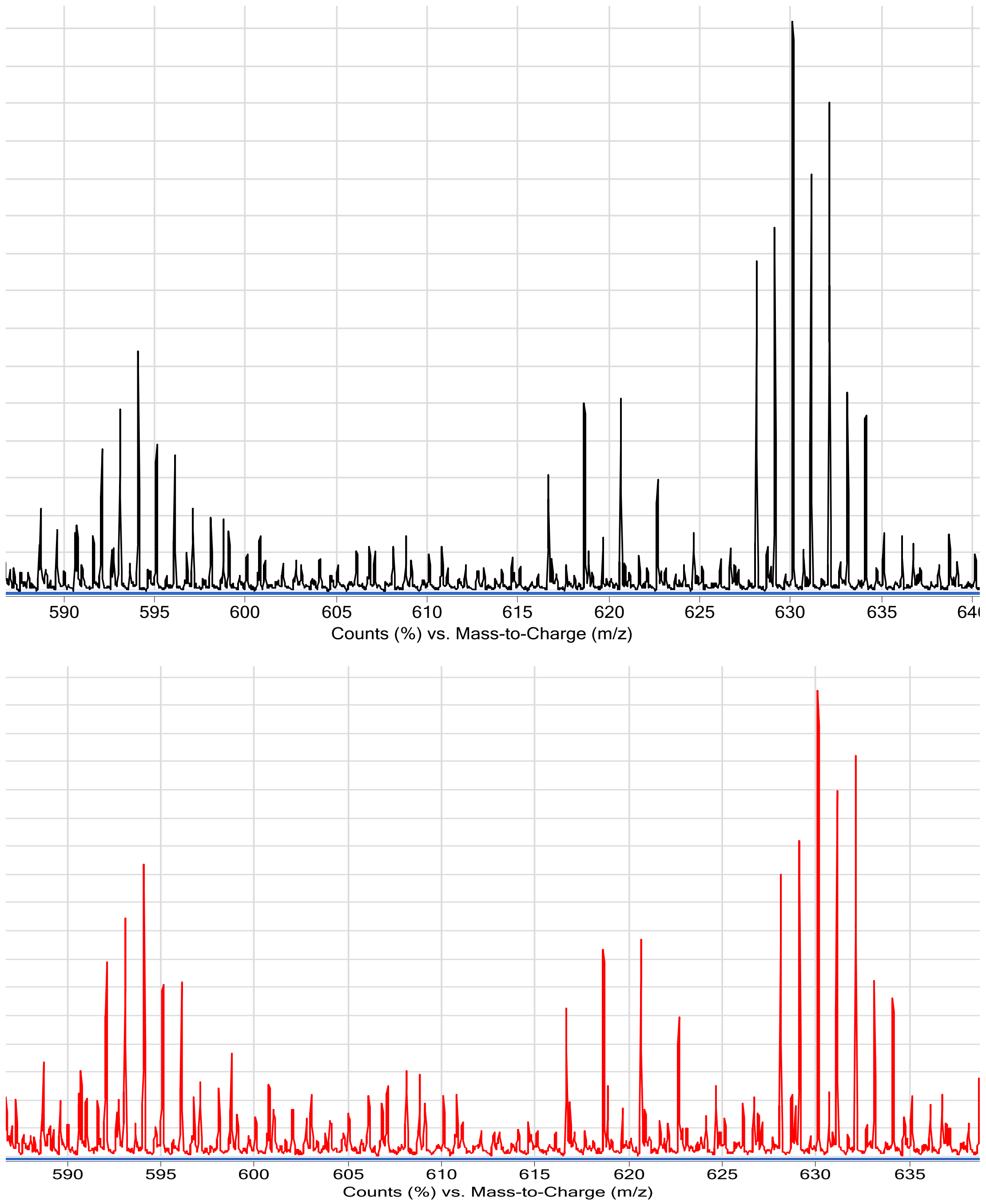

Mass spectrum of **3A** in physiological solution (0.9 % NaCl): top after dissolution; bottom after 48 h.

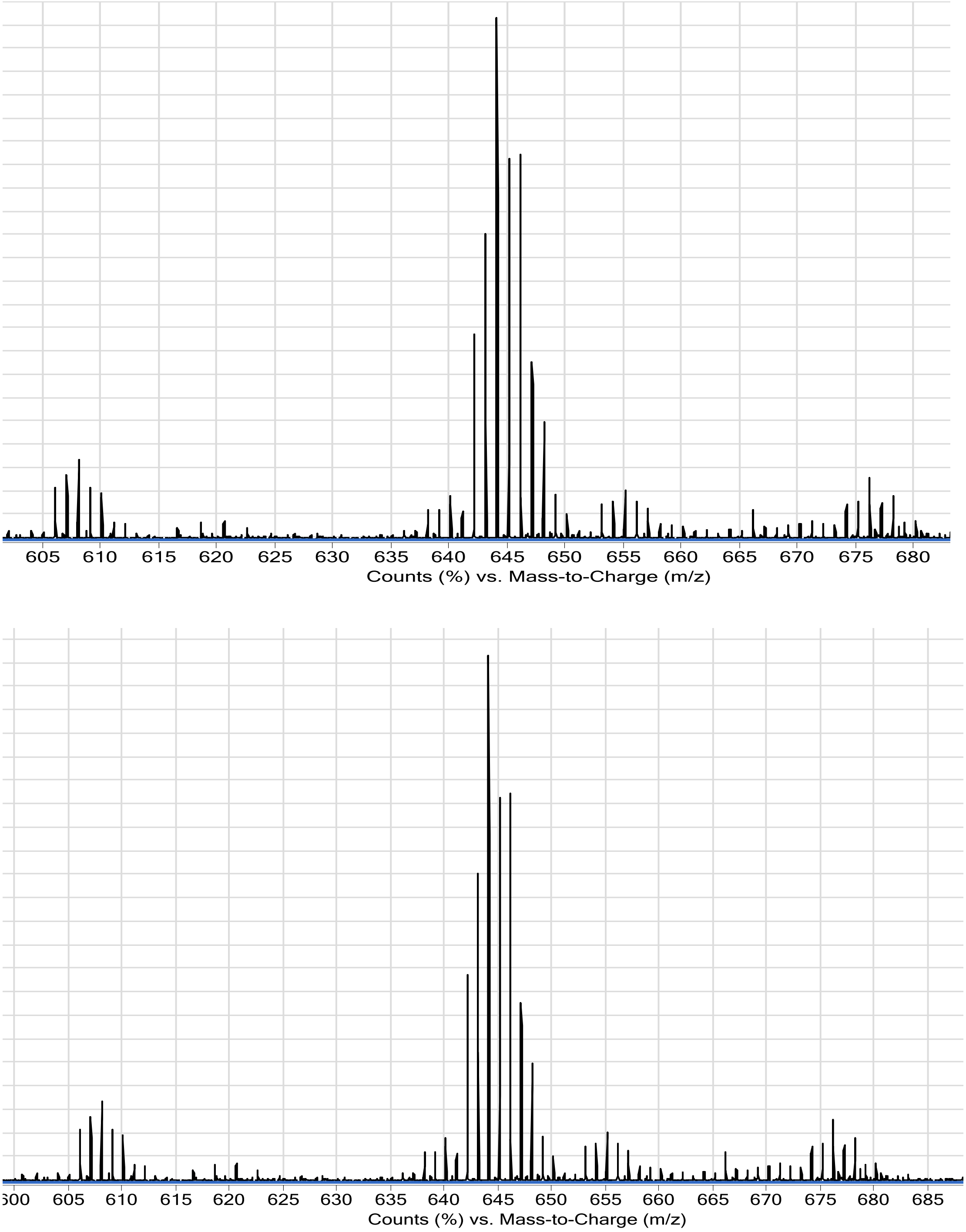

Mass spectrum of **3P** in physiological solution (0.9 % NaCl): top after dissolution; bottom after 48 h.

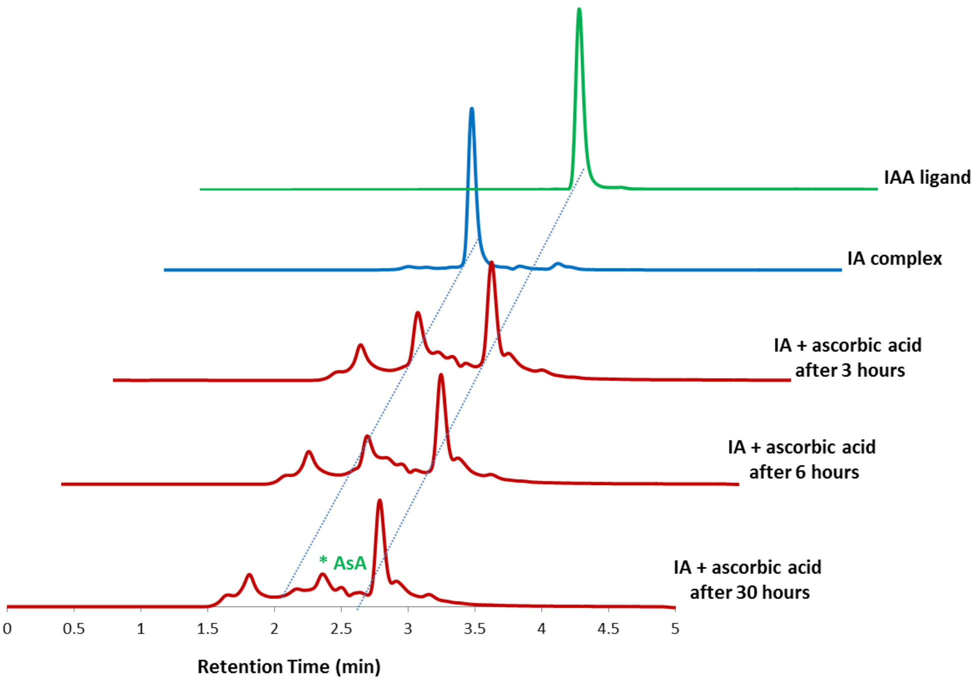

Figure S1: HPLC analysis of the reaction of **1A** with 10 eq. of ascorbic acid (AsA) at 37° C and pH 7. The chromatogram of the free ligand (IAA) is shown for comparison purposes.

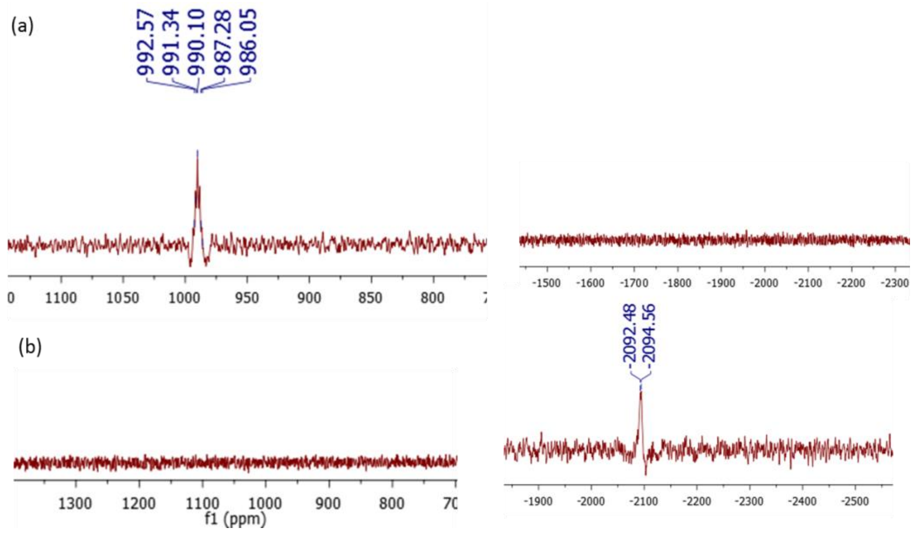

Figure S2. ^{195}Pt NMR spectra of (a) complex **1A** and (b) after reacting **1A** with ascorbic acid for 30 h at 37 °C. Zooms of the 600 to 1400 and -2300 to -2000 ppm regions are shown.

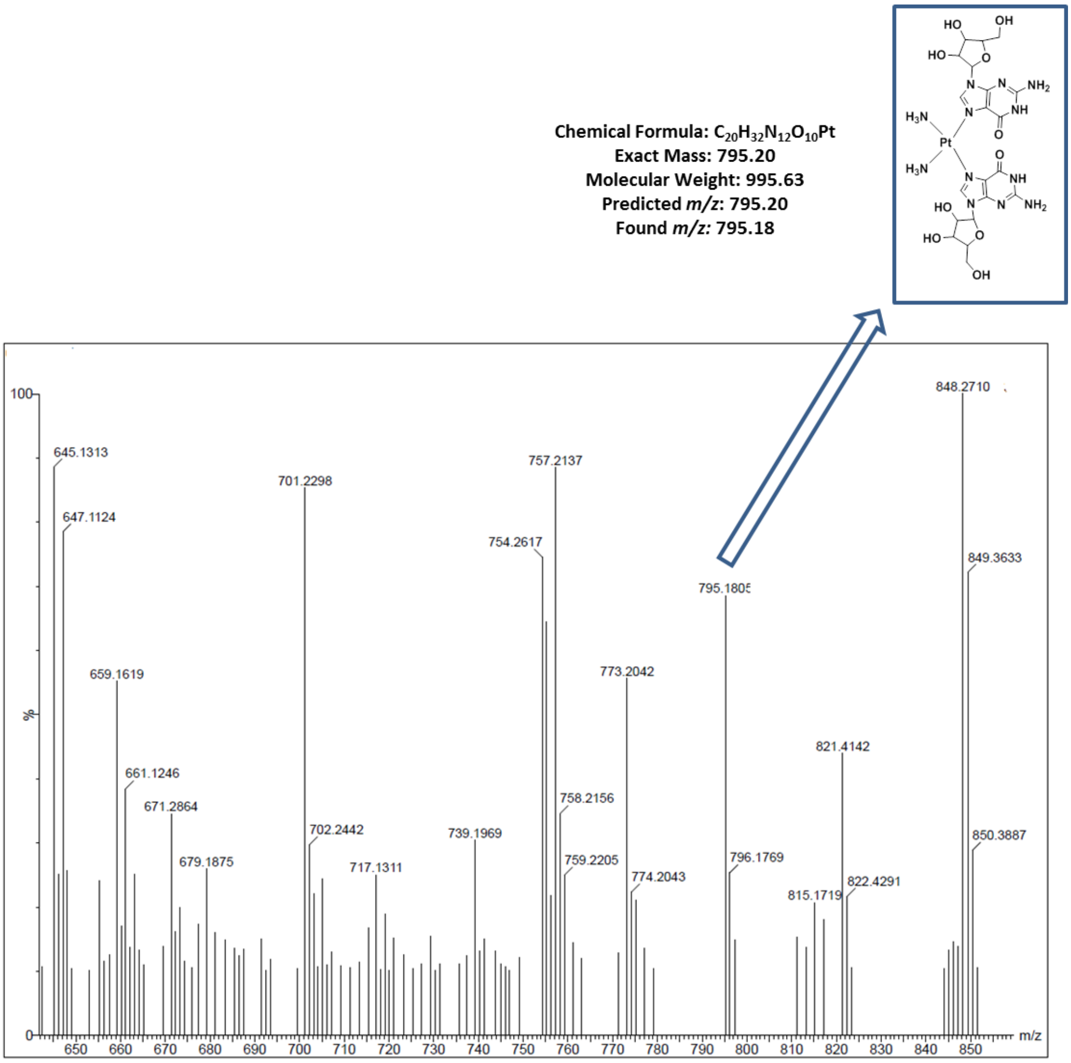

Figure S3: MS-ESI spectrum of the Pt-GG adduct obtained by the reaction of **1P** and guanosine in the presence of sodium ascorbate. The isotopic peak pattern confirms the presence Pt in the Pt-GG adduct.

A

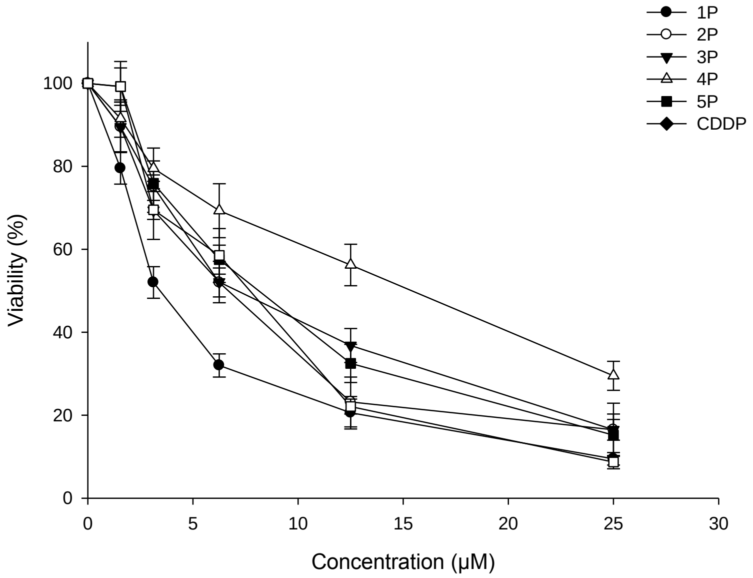

B

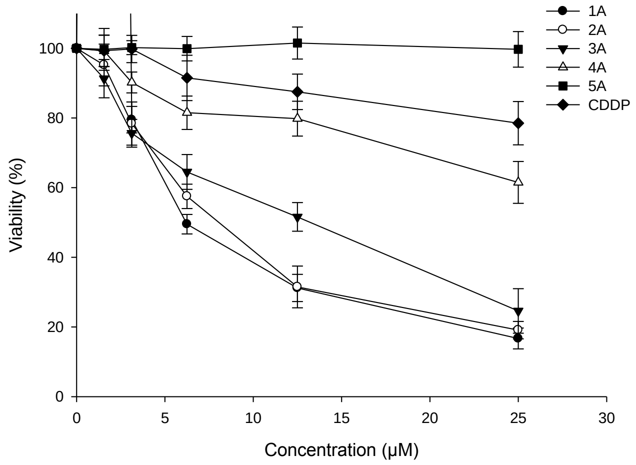

Figure S4. A375 cells (5×10^{3} mL^{-1}) were treated for 24 h with increasing concentrations of indole propionic acid Pt(IV) (panel A) or indole acetic acid (panel B) Pt(IV) complexes. The cytotoxicity was assessed by the MTT test. Data are the means of three independent experiments. Error bars indicate SD.
